# FLT3‐ITD mutations in acute myeloid leukaemia – molecular characteristics, distribution and numerical variation

**DOI:** 10.1002/1878-0261.12961

**Published:** 2021-05-02

**Authors:** Caroline Engen, Monica Hellesøy, Tim Grob, Adil Al Hinai, Atle Brendehaug, Line Wergeland, Siv Lise Bedringaas, Randi Hovland, Peter J. M. Valk, Bjørn T. Gjertsen

**Affiliations:** ^1^ Department of Clinical Science Centre for Cancer Biomarkers CCBIO University of Bergen Norway; ^2^ Haematology Section Department of Medicine Haukeland University Hospital Helse Bergen HF Norway; ^3^ Department of Haematology Erasmus University Medical Centre Rotterdam The Netherlands; ^4^ Department of Medical Genetics Haukeland University Hospital Helse Bergen HF Norway; ^5^ Department of Biosciences University of Bergen Norway

**Keywords:** acute myeloid leukaemia, *FLT3*‐ITD, length mutation, outcome, prognosis

## Abstract

Recurrent somatic internal tandem duplications (ITD) in the FMS‐like tyrosine kinase 3 (*FLT3*) gene characterise approximately one third of patients with acute myeloid leukaemia (AML), and *FLT3*‐ITD mutation status guides risk‐adapted treatment strategies. The aim of this work was to characterise *FLT3*‐ITD variant distribution in relation to molecular and clinical features, and overall survival in adult AML patients. We performed two parallel retrospective cohort studies investigating *FLT3*‐ITD length and expression by cDNA fragment analysis, followed by Sanger sequencing in a subset of samples. In the two cohorts, a total of 139 and 172 mutant alleles were identified in 111 and 123 patients, respectively, with 22% and 28% of patients presenting with more than one mutated allele. Further, 15% and 32% of samples had a *FLT3*‐ITD total variant allele frequency (VAF) < 0.3, while 24% and 16% had a total VAF ≥ 0.7. Most of the assessed clinical features did not significantly correlate to *FLT3*‐ITD numerical variation nor VAF. Low VAF was, however, associated with lower white blood cell count, while increasing VAF correlated with inferior overall survival in one of the cohorts. In the other cohort, ITD length above 50 bp was identified to correlate with inferior overall survival. Our report corroborates the poor prognostic association with high *FLT3*‐ITD disease burden, as well as extensive inter‐ and intrapatient heterogeneity in the molecular features of *FLT3*‐ITD. We suggest that future use of FLT3‐targeted therapy could be accompanied with thorough molecular diagnostics and follow‐up to better predict optimal therapy responders.

## Graphical abstract

Internal tandem duplications (ITD) in the FMS‐like tyrosine kinase 3 (*FLT3*) gene is present in 20–30% of acute myeloid leukaemia (AML) patients. Here, we perform molecular profiling of *FLT3*‐ITD mutations in 263 AML patients. We provide a comprehensive overview of the heterogeneity and impact of FLT3‐ITD mutations by assessing various molecular features and the relationship with clinical features and overall survival.

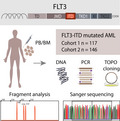

**Abbreviations**
(t‐)VAF: (total) variant allele frequency
AML: acute myeloid leukaemia
BM: bone marrow
FLT3: FMS‐like tyrosine kinase 3
HSCT: haematopoietic stem cell transplantation
ITD: internal tandem duplication
LM: length mutation
PB: peripheral blood
WBC: white blood cell

## 1 Introduction

The FMS‐like tyrosine kinase 3 (*FLT3*) is the single most frequently somatically mutated gene in AML [1, 2, 3]. Most *FLT3* mutations described result in aberrant gene products, as well as functionally deviating cell behaviour, in both *in vitro* [4, 5, 6] and *in vivo* models [7, 8]. *FLT3* internal tandem duplications (*FLT3*‐ITD), detected in 20–30% of AML patients, have repeatedly been shown to correlate with prediction of disease relapse as well as with inferior overall survival [9, 10, 11]. Consequently, *FLT3*‐ITD mutation status has become a well‐established prognostic biomarker in AML [12, 13]. The relationship between *FLT3*‐ITD mutation status and outcome is also influenced by *FLT3*‐ITD mutation load [11, 14, 15, 16, 17, 18, 19, 20], a feature which was recently included in the European Leukaemia Net (ELN) 2017 updated risk stratification guidelines [12]. *FLT3*‐ITD targeted therapy has confirmed *FLT3*‐ITD mutations as leukemic drivers as well as possible therapeutic targets both in model systems [21, 22] and in AML patients [23, 24, 25].

Despite two decades of accumulating data, the utility of FLT3‐targeting therapeutics has provided limited benefit both as monotherapy and in combination therapy [23, 25]. Results from the RATIFY trial, a large international multicentre phase III trial, recently demonstrated a 7.1% increase in 4‐year overall survival and a 21% relative risk reduction in patients treated with the broadly acting kinase inhibitor midostaurin as maintenance therapy [25]. QuANTUM‐R, a randomised controlled phase III trial, demonstrated a modest increase in overall survival in refractory or relapsed AML treated with the FLT3‐specific inhibitor quizartinib as monotherapy, where median survival was 6.2 months in the exploratory arm compared to 4.7 months in the control arm [23]. In the phase III ADMIRAL trial, comparing gilteritinib treatment to salvage chemotherapy in FLT3‐ITD mutated relapsed/refractory AML patients, the median overall survival was moderately improved from 5.3 months in the control arm to 9.3 months in the experimental arm [26]. FLT3‐targeted therapy has also been shown to improve long‐term outcome when administered as maintenance therapy of *FLT3* mutated AML after allogeneic stem cell transplantation [27, 28], a disease state characterised by low tumour burden and anti‐leukemic immunological mechanisms.

Although the poor risk association of *FLT3*‐ITD mutations in AML is well established, the impact of the significant inter‐ and intrapatient heterogeneity in various molecular features of ITDs is still unclear. Numerical variation of *FLT3*‐ITD mutations, duplication length, duplication sequence and the insertion/duplication integration site are all characteristics of *FLT3*‐ITD mutated AML that have been shown to influence disease outcome [10, 15, 29, 30, 31, 32, 33]. However, no clear consensus currently exists regarding the significance of these features. Furthermore, the molecular mechanisms underlying this diversity is unknown and the strength and direction of these various associations are conflicting [5, 14, 15, 16, 17, 20, 32, 34, 35, 36, 37, 38]. Thus, understanding more about the heterogeneity and complexity of *FLT3* mutations in AML may reveal relationships that could inform future efforts directed at improving FLT3‐targeted approaches. In this report, we present results from retrospective molecular profiling of *FLT3*‐ITD mutations in a total of 263 AML patients. We provide a comprehensive overview of the heterogeneity and impact of FLT3‐ITD mutations in AML by assessing the numerical variation, variant allele distribution and the relationship with clinical features as well as with *FLT3*‐ITD molecular characteristics like length, sequence and integration site correlated to overall survival in two independent AML cohorts.

## 2 Materials and methods

### 2.1 Patients

This is a retrospective study assessing adult patients with AML (M3 excluded) between the age of 15 and 80 included and treated on various study protocols of the Dutch‐Belgian Haemato‐Oncology Cooperative Group (HOVON) and the Leukaemia Group of the Swiss Group for Clinical Cancer Research (SAKK) during the period 1987–2013. Our analysis was restricted to patients with predetermined *FLT3*‐ITD mutation and availability of cDNA samples for further molecular characterisation. This comprised patients treated in the protocols HO04, HO04a [39], HO29 [40, 41], HO42 [42, 43], HO43 [44] and HO102 [45], respectively. The patients were split into two cohorts (C1 and C2) based on the timing of *FLT3*‐ITD status determination; retrospectively or prospectively. C1 constitutes patients from HO04, HO04a, HO29, HO42 and HO43 (1987–2006) while C2 comprises patients from HO102 (2009–2013). Additional information about the individual trials can be obtained at http://www.hovon.nl. A selection of patients in C1 has previously been reported on with regards to *FLT3*‐ITD status [46] and crude ratios with focus on white blood cell (WBC) counts and prognostic association [47].

### 2.2 Ethics

Clinical trials were approved by local ethics committees and performed in accordance with the Declaration of Helsinki. All participants signed and submitted written informed consent at trial inclusion. The consent covered use of biological material for research not directly related to the clinical trial.

### 2.3 Sample processing

Sampling and data gathering were performed as previously described [45, 48, 49]. In short, bone marrow (BM) and/or peripheral blood samples were collected at time of study inclusion. The mononuclear cell fraction was isolated by Ficoll‐Hypaque centrifugation and the cells were cryopreserved and stored at the Erasmus University Medical Centre, Rotterdam, until further processing.

### 2.4 DNA fragment analysis by capillary electrophoresis

Length mutations in the juxtamembrane region of the *FLT3* gene were validated and characterised by DNA fragment analysis by capillary electrophoresis. The procedure was performed independently for the C1 and C2 cohorts at two separate centres. Samples in C2 were analysed as previously described [45]. For samples in C1, the concentration of complementary DNA (cDNA) and genomic DNA (gDNA) was quantified and normalised to approximately 20 ng·μL^−1^ adjusted by ddH2O by NanoDrop 1000 (ThermoFisher). 1 μL of each sample was subsequently amplified by polymerase chain reaction (PCR), according to standard protocols. We used AmpliTaq Gold 360 Master Mix (Applied Biosystems, Waltham, MA, USA. Cat nr 4398881) and two discrete primer sets; one set for the cDNA reactions and one set for the gDNA reactions, respectively: 11F [6FAM]GCAATTTAGGTATGAAAGCCAGC and e15R1 CATAAGCTGTTGCGTTCATCAC, and i13F – [6FAM]GCAGAACTGCCTATTCCTAACTG and e15R1 – CATAAGCTGTTGCGTTCATCAC (desalted, Sigma‐Aldrich, St. Louis, MO, USA). The PCRs, performed on a thermic cycler (GeneAmp PCR System 9700, Applied Biosystems/S1000 Thermal Cycler, Bio‐Rad, Hercules, CA, USA), were run according to the following profile: initialisation at 95 °C for 10 min permitting enzyme activation. Enzyme driven DNA replication was performed for 29 thermal cycles under the conditions of 94 °C for 30 s, 62 °C for 30 s and 72 °C for 1 min, allowing denaturation, annealing and elongation. The reaction was finalised by a finishing elongation step at 72 °C for 7 min, before the samples were cooled to 4 °C until further processing. The amplified fragments were mixed with a size marker (GeneScan–500 ROX, Applied Biosystems) and HiDi Formamid (Applied Biosystems) according to manufactures instructions and separated by size using capillary electrophoresis (ABI 3100 Genetic Analyzer (POP4 polymer), Applied Biosystems, Thermo Fisher Scientific, Waltham, MA, USA). Data were analysed in Peak Scanner (Applied Biosystems) in accordance with developers’ guidelines, determining fragment size relative to an internal control. All analyses were performed in triplicates.

### 2.5 Cloning

cDNA was amplified by PCR as described above, but run for a total of 35 cycles and using a forward primer without a 6FAM label. The amplified PCR products were subsequently cloned using TOP10 chemical competent cells according to the TOPO TA cloning manual (Invitrogen, Thermo Fisher Scientific, Waltham, MA, USA). Positive colonies were directly PCR‐amplified and fragments were analysed by fragment analysis by capillary electrophoresis, as described above.

### 2.6 Sanger sequencing

Positive clones (defined as PCR fragments larger than the estimated length product of the wild‐type *FLT3* fragment) were re‐amplified using a forward primer without the 6FAM label and further purified using ExoZap‐IT (Applied Biosystems), or illustra Exoprostar 1‐step (VWR, Radnor, PA, USA) and PCR‐amplified for sequencing. BigDye v1.1 Terminator cycle sequencing kit (Applied Biosystems) was used to perform direct sequencing and the products were analysed on an ABI 3730 Genetic Analyzer (POP7 polymer), (Applied Biosystems) according to the manual. The sequences were analysed using FinchTV (Geospiza Inc., Seattle, WA, USA).

### 2.7 Statistical methods

Peaks larger than the peak representing the *FLT3* wild‐type product, identified in all three technical replicates, were considered to represent probable individual *FLT3*‐ITD mutations. Fragment length of the PCR product was calculated as the mean value of three replicates. The relationship between the wild‐type peak and additional peaks in the sample was calculated as variant allele frequencies (VAF) (individual fragment/sum of fragments in the sample). A total VAF (t‐VAF) was calculated for each sample, representing the load of *FLT3*‐ITD mutants (sum aberrant fragments/sum of all fragments). A VAF of 0 indicates no detected mutation, whereas a VAF of 1 indicates loss of the wild‐type allele in all cells.

We performed descriptive and univariate analyses to characterise the cohorts based on disease‐related variables. The Wilcoxon signed‐rank test/Mann–Whitney (non‐parametric) test was applied for pairwise comparison of continuous variables. For comparison of categorical variables, we performed 2 × 2 tables and applied the 2‐sided Fisher exact test. Pearson correlation was used to test relationships between continuous variables. For comparison of patient, disease and survival differences with respect to *FLT3*‐ITD t‐VAF, *FLT3*‐ITD length and *FLT3*‐ITD insertion site, the variables were dichotomised in agreement with optimally selected cutpoints calculated by maximally selected rank statistics. All statistical tests comparing clinico‐pathological features across groups are summarised in the supplementary tables (Tables S1–S6). Notably, not all tests comprised the full sample set due to incomplete data. Median follow‐up was estimated by the reverse Kaplan–Meier method. Overall survival was calculated by the Kaplan–Meier method and visualised by Kaplan–Meier plots. The 2‐sided log‐rank test was applied to compare the Kaplan–Meier estimates. Logistic regression analysis was applied to identify factors most closely associated with overall survival. The multivariate Cox proportional hazards regression model included age, sex, and WBC count in addition to *FLT3*‐specific variables. Statistical significance was defined as *P*‐value ≤ 0.05. All statistical calculations and graphical representations were performed in r‐studio (version 1.1.453) and r (version 3.5.0) [50]. Supplementary tables include *P*‐values adjusted for multiple testing calculated by the Benjamini and Hochberg method [51].

## 3 Results

### 3.1 Cohort composition

Cohorts C1 (1987–2006) and C2 (2009–2013) comprise 432 and 625 treatment naïve non‐M3 AML patients, respectively. The two cohorts are significantly different with regard to some central baseline features, summarised in Table S1. Notably, the patient population in C1 is significantly younger (46 years vs 53 years, *P* < 0.0001) and the median WBC count is significantly higher (29.3 vs 9.8, *P* < 0.0001). Conversely, the proportion of individuals that received allogeneic haematopoietic stem cell transplantation (allo‐HSCT) was substantially higher in C2 compared to C1 (C1: 127/432 vs C2: 313/624, *P* < 0.0001). There was also a slight asymmetry related to the fraction of individuals receiving autologous HSCT (auto‐HSCT) (60/432 vs 61/624, *P* = 0.0490). C1 is confined by available sample material and is therefore enriched for patients with high disease burden compared to C2, while C2 is delineated by protocol inclusion, accounting for the differences in WBC counts. Disparities in treatment are largely due to the temporal separation of these two cohorts.

Of the 432 and 625 patients included in the initial screen for *FLT3*‐ITD mutations, a total of 117 (27.1%) and 146 (23.4%) *FLT3*‐ITD‐positive samples were identified in C1 and C2, respectively. There was no significant difference in the fraction of *FLT3*‐ITD‐positive samples between the two cohorts.

### 3.2 *FLT3*‐ITD patient characteristics

In both cohorts, there was a tendency towards more frequent *FLT3*‐ITD mutations in females compared to males, although not significant (C1 F:67/215 vs M:50/365, *P* = 0.07. C2 F: 74/273 vs M:72/352, *P* = 0.06). *FLT3*‐ITD mutations were associated with high BM blast percentage at time of diagnosis (C1 *P* < 0.0001) and with high WBC counts (C1 and C2 *P* < 0.0001). *FLT3*‐ITD mutations were identified across all defined FAB categories but were comparatively enriched in M1 (C1 *P* = 0.08, C2 *P* = 0.048) and conversely underrepresented in M0 (C1 *P* = 0.08, C2 *P* = 0.01), although only significant in C2. *FLT3*‐ITD mutations were most common in patients characterised by normal karyotype (C1 and C2 *P* < 0.0001) and rare in core‐binding factor (CBF) leukaemia (C1 *P* < 0.0001 and C2 *P* = 0.006) as well as in individuals with complex karyotype (C1 *P* = 0.004 and C2 *P* < 0.0001) and inv16 (C1 *P* < 0.0001 and C2 *P* = 0.047). *FLT3*‐ITD mutations frequently co‐occurred with *NPM1* (C1 and C2 *P* < 0.0001) and *DNMT3A* mutations (C1 *P* = 0.039 and C2 *P* = 0.003) and were mutually exclusive with *NRAS* mutations (C1 *P* < 0.0001). *FLT3*‐ITD mutations rarely co‐occurred with *ASXL1* (C1 *P* = 0.039 and C2 *P* = 0.004) and TP53 (C2 *P* = 0.001) mutations (Table S2C1,C2).

### 3.3 *FLT3*‐ITD variant allele distribution

**Fig. 1:**
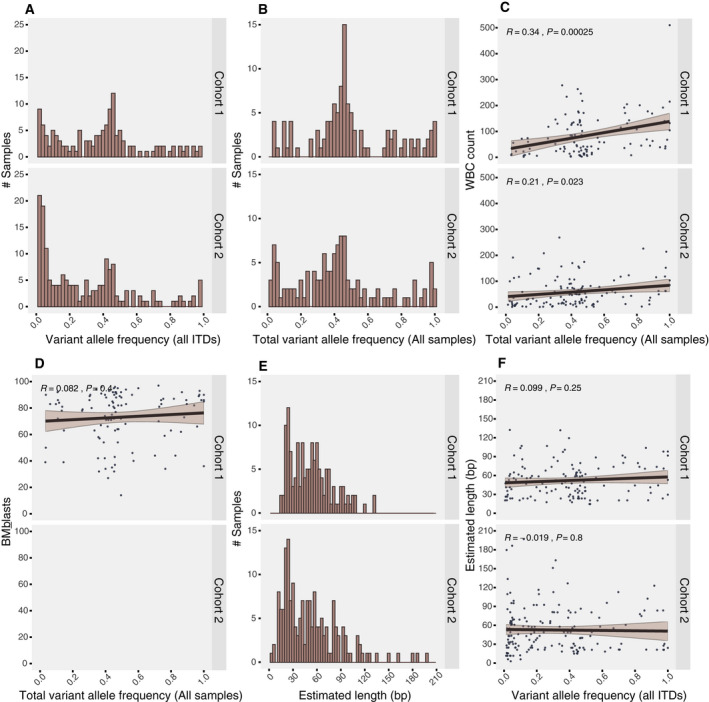
Variant allele frequency and correlation to baseline clinical features. (A) Distribution of variant alle frequency (VAF) of all putative internal tandem duplications (ITDs) as identified and estimated by cDNA fragment analysis by capillary electrophoresis (Cohort1: *n* = 139, Cohort2: *n* = 172). (B) Distribution of total VAF (t‐VAF) in all characterised *FLT3*‐ITD positive samples (Cohort 1: *n* = 111, Cohort 2: *n* = 123). (C) Correlation analysis of t‐VAF and white blood cell (WBC) count (Cohort 1: *n* = 111, *R* = 0.34, *P* = 0.00025, Cohort2: *n* = 123, *R* = 0.21, *P* = 0.023). (D) Correlation analysis of t‐VAF and bone marrow (BM) blast percentage (Cohort 1: *n* = 108, *R* = 0.082, *P* = 0.4, Cohort 2: data not available). (E) Distribution of putative ITD length as identified and estimated by complementary DNA (cDNA) fragment analysis by capillary electrophoresis (Cohort 1: *n* = 139, Cohort 2: *n* = 172). (F) Correlation analysis of VAF and *FLT3*‐ITD length as identified and estimated by cDNA fragment analysis by capillary electrophoresis (Cohort 1: *n* = 139, *R* = 0.099, *P* = 0.25, Cohort 2: *n* = 172, *R* = −0.019, *P* = 0.8). Strength of linear associations was assessed by Pearson correlation coefficient. The shaded area in correlation curves indicates the 95% confidence interval.

The relationship between *FLT3*‐ITD variant alleles and wild‐type alleles assessed in gDNA is a direct function of the cellular distribution of *FLT3*‐ITD mutated cells in the sample, while the same relationship in cDNA is a function of the expression of the various *FLT3* alleles. It is not clear whether the wild‐type and mutated alleles are equally expressed. We therefore correlated the VAF estimated from cDNA and gDNA in 84/116 samples from C1 and identified 95 corresponding ITDs in 82 patients (Fig. S1A). The overall correlation of VAF in cDNA and gDNA was very strong (*n* = 95, *R* = 0.96, *P* < 2.2e‐16) (Fig. 1B). Based on this relationship and the availability of cDNA for most samples, we preceded with molecular assessment of *FLT3*‐ITDs with respect to length and the relationship with the wild‐type allele in cDNA for 116/117 cases in C1 and 117/146 cases and C2. In C2, we included six cases where cDNA was missing, and the analysis was performed using gDNA.

### 3.4 Number of distinct *FLT3*‐ITDs per patient

In C1, we verified at least one mutant allele in 111/116 cases, identifying a total of 139 mutant alleles. A single mutant allele was identified in 78% (87/111) of the samples while 22% (24/111) had more than one mutant allele of varying size, including 21 samples with two mutant alleles, two samples with three mutant alleles and one sample with four mutant alleles. Across the 123 samples in C2, we identified a total of 172 mutant alleles. One mutant allele was identified in 72% (88/123) while 28% (35/123) had plural mutant alleles, including 24 samples with two mutant alleles, eight samples with three mutant alleles and three samples with four mutant alleles. There was no significant relationship between patients characterised by a single mutant allele and patients characterised by multiple mutant alleles with respect to sex, age, WBC count, BM blast percentage, FAB classification or cytogenetics. Furthermore, there were no differences in other assessed mutations or *FLT3*‐specific features like VAF or length in the two cohorts (Table S3C1,C2).

### 3.5 *FLT3*‐ITD variant allele fraction

Across all mutant alleles (C1 = 139 and C2 = 172), the median VAF was 0.40 (range: 0.006–1) and 0.22 (range: 0.013–0.999) in C1 and C2, respectively (Fig. 1A). Summarising the total *FLT3*‐ITD VAF (t‐VAF) in both cohorts with plural mutant alleles (C1 = 111 and C2 = 123), the median t‐VAF was 0.4559 (range: 0.0327–1.0000) and 0.4059 (range: 0.0129–1.0000), respectively (Fig. 1B). This suggests that the variation between C1 and C2 when comparing all mutant alleles may be attributed to detection of more low VAF ITDs in C2 compared to C1.

Next, we assessed the association between VAF and baseline clinical features. We dichotomised VAF by maximally rank statistics, which produced a biphasic curve with the optimal cutpoint indicated at a VAF of 0.7, but with a peak of comparable size at 0.3. Thus, we present data using both cutpoints. In C1, 15% (17/111) of samples had a t‐VAF < 0.3 while 24% (27/111) had a t‐VAF ≥ 0.7. In C2, 32% (39/123) of the samples had a t‐VAF < 0.3, while 16% (20/123) of samples had a t‐VAF ≥ 0.7. In both cohorts, we found that increasing t‐VAF was associated with higher WBC count (C1 *n* = 111, *R* = 0.341, *P* = 0.0003 and C2 *n* = 123, *R* = 0.206, *P* = 0.02) (Fig. 1C), but not with higher blast percentage (only available in C1; *n* = 109, *R* = 0.0818, *P* = 0.4) (Fig. 1D). Age, platelet count and karyotype were not associated with variation of t‐VAF. Comparing t‐VAF between samples positive and negative for the various variants we assessed for, we found that lower t‐VAF was characteristic of *CEBPA*‐double mutated samples in C1 (*P* = 0.031), while in C2 *FLT3*‐ITD t‐VAF was higher in *DNMT3A* mutated samples (*P* = 0.005) (Tables S4C1,C2 and S5C1,C2).

### 3.6 Base pair length of the *FLT3*‐ITD

Next, we assessed *FLT3*‐ITD length, defined as the number of additional base pairs compared to wild‐type *FLT3*. The median estimated ITD length across all patients (*n* = 139) was 51 bp (range: 15–132) in C1 and 45 bp (range: 3–198) in C2 (*n* = 172) (Fig. 1E). The median ITD length of the mutated allele with the highest VAF (LM1) in each patient sample was 51 bp (range: 15–132) in both C1 (*n* = 111) and C2 (*n* = 123) (range: 3–186).

Focusing on LM1, we dichotomised the ITD length variable for comparison with baseline features. Assessment with maximally rank statistics suggested a division at 50 bp. We found that 50% (55/111) of samples in C1 had a LM1 shorter than 50 bp. The same was true for 54% (66/123) of samples in C2. We did not find statistically significant correlations between *FLT3*‐ITD length and any clinical features. In C1, we did, however, identify an association between shorter ITDs and *DNMT3A* mutations (< 50:25/55 vs ≥ 50:10/56, *P* = 0.022), although this relationship was not significant in C2 (Table S6C1,C2). The estimated ITD length did not correlate with the respective VAF (C1: *R* = 0.099, *P* = 0.25. C2: *R* = 0.019, *P* = 0.8) (Fig. 1F).

**Fig. 2:**
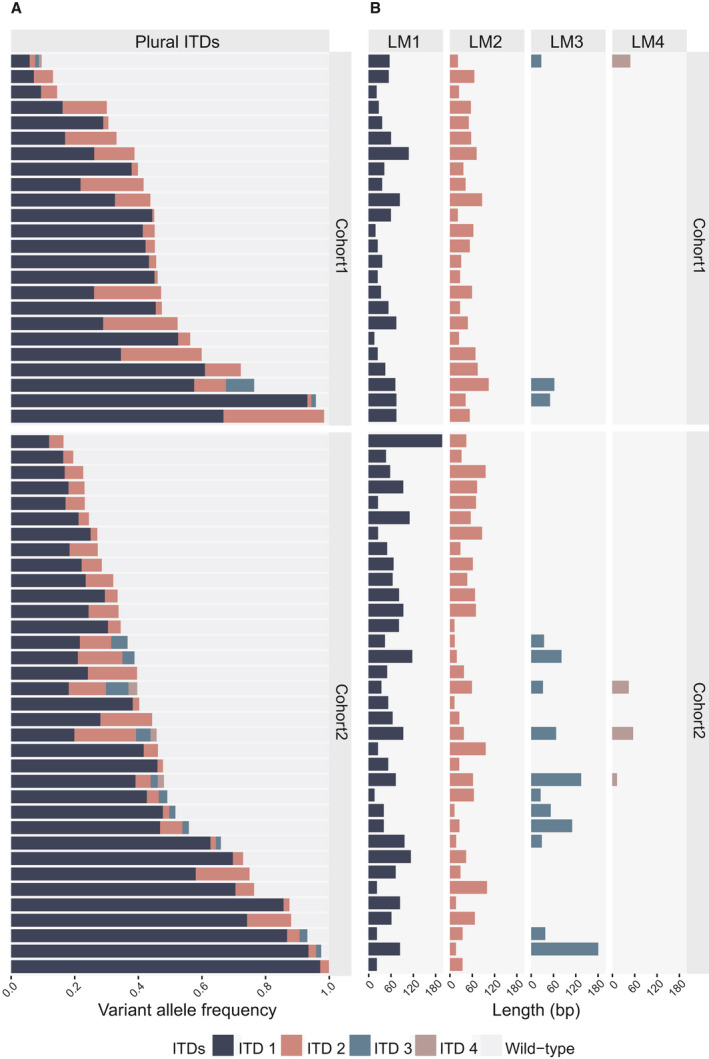
*FLT3*‐ITD variant allele frequency and length in patients with multiple ITDs. (A) Graphical representation of the variant allele frequency (VAF) of various internal tandem duplications (ITDs) in samples where more than one *FLT3*‐ITD was indicated by fragment analysis, in cohort 1 (*n* = 24, upper panel) and cohort 2 (*n* = 35, lower panel). Each bar represents a single patient sample, where each individual ITD is colour‐coded. LM1‐4 indicates individual ITDs numbered and ordered by descending VAF. (B) Length of the respective ITDs in base pairs (bp).

In samples exhibiting plural ITDs, we observed no clear pattern in the size (as assessed by VAF) of LM1 in relation to the remaining mutant alleles of lower VAF; some patients were characterised by one dominating ITD, while other patients displayed co‐existence of multiple similarly sized leukemic cell populations harbouring distinct *FLT3*‐ITDs (Fig. 2A). In C1, the ITD length distribution of LM1 across patients did not significantly differ from the ITD length distribution of mutant alleles with lower VAF (24 LM1: 42 bp vs 28 LM‐non‐LM1: 51 bp, *P* = 1). However, we observed a tendency towards shorter length of the mutant alleles with lower VAF in C2 (35 LM1: 60 bp vs 49 LM‐not‐LM1: 42 bp. *P* = 0.053). In C1, 58% of the second largest mutant allele (LM2) exceeded the length of LM1 (14/24) while the same was true in 28% of cases is C2 (10/35) (Fig. 2B).

### 3.7 *FLT3*‐ITD sequence and putative motif characteristics

**Fig. 3:**
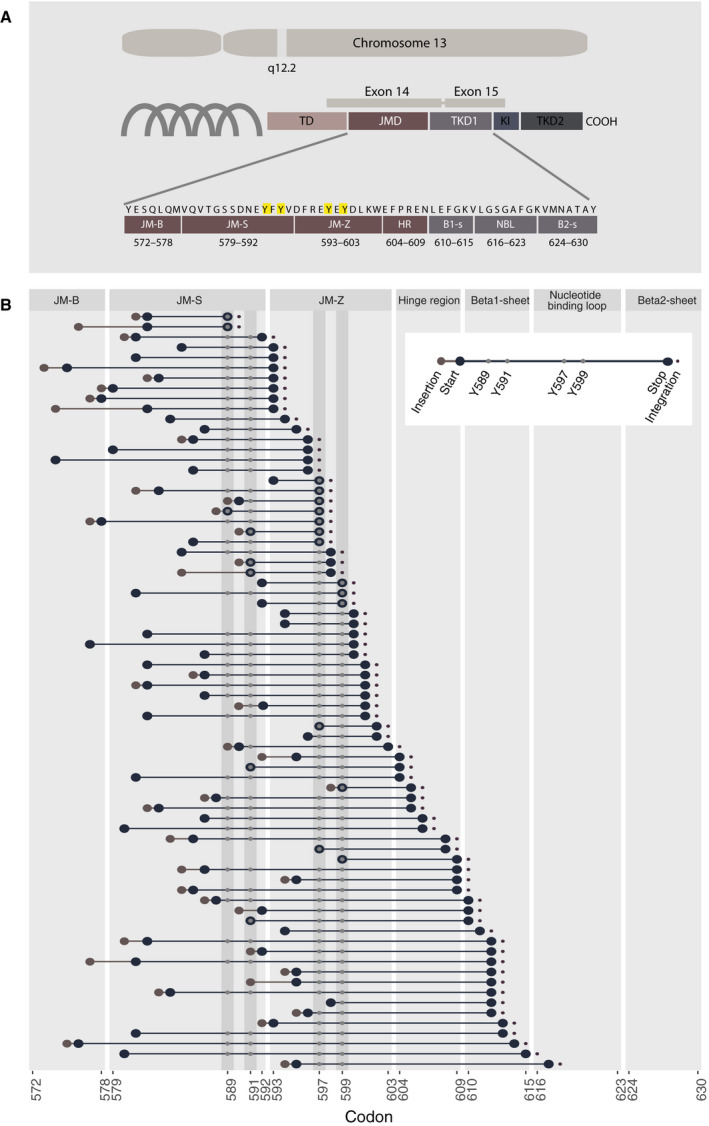
*FLT3* protein structure and length and position of individual ITDs. (A) *FLT3* gene chromosomal position and gene encoding protein structure with focus on the juxtamembrane domain and the tyrosine kinase domain 1 covering exon 14–15 in accordance with functional regions as presented by [20]. B1‐s, Beta1‐sheet; B2‐s, Beta2‐sheet; HR, hinge region; JM‐B, Juxtamembrane binding motif; JMD, Juxtamembrane domain; JM‐S, Juxtamembrane Switch motif; JM‐Z, Juxtamembrane zipper motif; KI, Kinase insert; NBL, Nucleotide‐binding loop; TD, Transmembrane domain; TKD, Tyrosine kinase domain 1; TKD2, Tyrosine kinase domain 2. (B) Graphical representation of all *FLT3*‐ITDs sequenced (Cohort1: *n* = 74), indicating length of insertion, length and position of duplicated sequence according to the amino acid sequence of *FLT3* as well as integration site. Each line indicates individual ITDs. The x‐axis indicates the corresponding codons of FLT3 protein sequence.

**Fig. 4:**
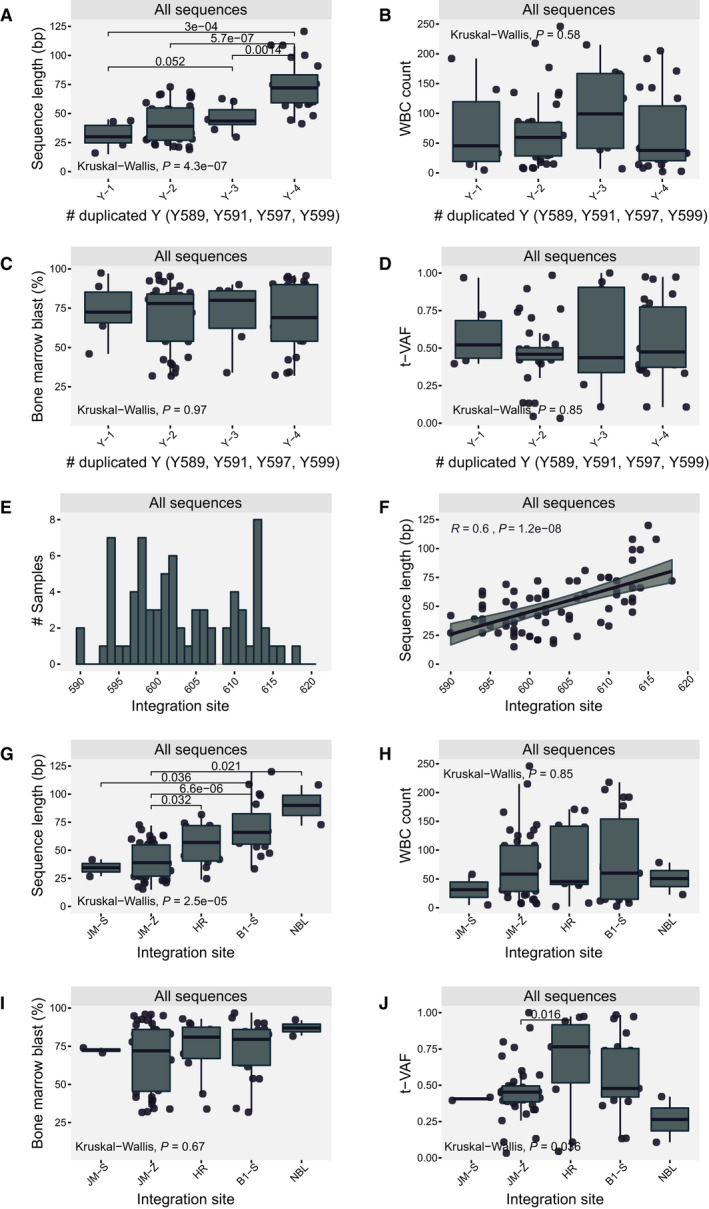
Molecular features of *FLT3*‐ITDs and association with clinical features. (A) Relationship between the number of duplicated tyrosine residues and the internal tandem duplication (ITD) sequence length in base pairs (bp). (B) Relationship between the number of duplicated tyrosine residues and the white blood cell (WBC) count. (C) Relationship between the number of duplicated tyrosine residues and the bone marrow blast percentage. (D) Relationship between the number of duplicated tyrosine residues and the *FLT3*‐ITD variant allele frequency (t‐VAF). (E) Distribution of all *FLT3*‐ITDs with regard to insertion site. (F) Correlation analysis between *FLT3*‐ITD integration site and *FLT3*‐ITD length in bp. (G) Relationship between the integration site and the sequence length in bp. (H) Relationship between the integration site and the WBC count. (I) Relationship between the integration site and the bone marrow blast percentage. (J) Relationship between the integration site and the *FLT3*‐ITD variant distribution. Statistical significance between multiple groups was assessed by the Kruskal–Wallis test. Pair‐wise relationships assessed by the Wilcoxon rank‐sum test, and significant relationships with *P*‐values are indicated in the figure. All boxplots show interquartile range (25th to 75th percentile) with median indicated. Minimum and maximum values are indicated by error bars. Strength of linear associations was assessed by Pearson correlation coefficient. The shaded area in correlation curves indicates the 95% confidence interval.

Cloning and DNA sequence analysis was performed in 66/116 cDNA samples from C1, and the duplicated motifs were classified with regard to the number of duplicated tyrosine residues as well as integration site (illustrated in Fig. 3A). A total of 74 ITDs were characterised (Fig. 3B). We identified one sequence in each of 58 samples and two sequences in eight samples. In one sample (6366), we identified one ITD, one 6 bp insertion and one 12 bp deletion. 54% (40/74) of ITDs were preceded by insertions of varying length, with a median of 3 bp and a range up to 24 bp, all expected to result in altered amino acid sequence. Previous studies have demonstrated heterogeneity of the duplicated motif, with hardly two identical ITDs within a study population [14, 20, 34]. Further, the duplicated sequence can cover several functionally distinct entities of the gene. Despite this heterogeneity, there seem to be some highly conserved elements, which we confirm in our cohorts. All ITDs span at least one tyrosine residue from the tyrosine rich stretch Y591‐Y599 (YVDREYEY). We identified six ITDs that span a single tyrosine residue, 36 ITDs that span two tyrosine residues and eight and 24 spanned three and four tyrosine residues, respectively. The number of duplicated tyrosine residues correlated with ITD length (Fig. 4A), but no association was found with WBC counts, BM blast percentage or t‐VAF (Fig. 4B–D). We further assessed the ITD integration site in accordance with the functional structure of the FLT3 protein in line with previous reports [20, 35]. The position of the integration site strongly correlated with *FLT3*‐ITD length (*R* = 0.6, *P* < 0.001) (Fig. 4E,F). Analogously, integration region correlated with *FLT3*‐ITD length (Fig. 4G). We found that 28% (21/72) of sequences integrated within the tyrosine kinase domain 1 with 19 sequences located in the Beta sheet 1 and 2 in the nucleotide‐binding loop. The remaining sequences were located in the juxtamembrane domain, with two in the Switch motif of the juxtamembrane domain, 40 in the Zipper motif of juxtamembrane domain region and 11 in the hinge region. No association was found with WBC counts or BM blast percentage (Fig. 4H,I). Integration in the hinge region was associated with higher t‐VAF (Fig. 4J).

### 3.8 Outcome of *FLT3*‐ITD mutated AML patients

Next, we assessed the outcome of the *FLT3*‐ITD mutated patients in the two cohorts. The median follow‐up time was 113.7 months (95% CI 102.5–122.9) and 42.3 months (95% CI 40.9–43.7) in C1 and C2, respectively. During the treatment course, 24% (27/111) and 67% (82/123) of patients in C1 and C2 underwent allo‐HSCT, respectively. An additional 15 patients in C1 and 13 patients in C2 received an auto‐HSCT. In C1, *FLT3* mutation status was retrospectively assessed and did not influence treatment decisions, while in C2, individuals characterised as *FLT3*‐ITD mutated were usually recommended an allo‐HSCT in first complete remission if considered eligible. For survival analysis, however, no patients were censored at time of HSCT.

As expected from the composition of the two cohorts and their temporal separation, C2 has a significant superior survival with a median survival of 32.0 months (95% CI 27.4–41.8 months) as compared to 18.8 months (95% CI 16.0–24.7 months, *P* = 0.031) for C1. This was also true for the *FLT3* mutated patients, who had a median overall survival of 9.2 months (95% CI 8.11–1–5–5) in C1 (*n* = 111) and 17.5 months (95% CI 13.7–40.7) in C2 (*n* = 123) (*P* = 0.022).

**Fig. 5:**
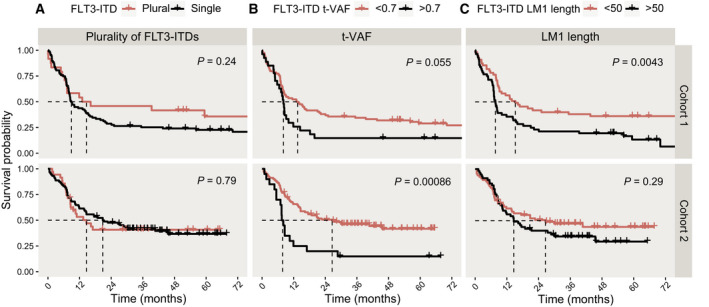
Survival analysis based on molecular features of *FLT3*‐ITD. Kaplan–Meier plots and log‐rank statistics of overall survival from study entry for *FLT3*‐ITD‐positive patients, separated by (A) detection of single (Cohort 1: *n* = 87, Cohort 2: *n* = 88) or plural *FLT3*‐ITDs (Cohort 1: *n* = 24, Cohort 2: *n* = 35) (Cohort 1 *P* = 0.24, Cohort 2 *P* = 0.79) (B) *FLT3*‐ITD variant distribution lower than 0.7 (Cohort 1: *n* = 84, Cohort 2: *n* = 103) or equal/higher than 0.7 (Cohort 1: *n* = 27, Cohort 2: *n* = 20) (Cohort 1 *P* = 0.055, Cohort 2 *P* = 0.00086) and (C) length of the *FLT3*‐ITD with highest variant allele frequency (VAF) below (Cohort 1: *n* = 55, Cohort 2: *n* = 66) or equal/above (Cohort 1: *n* = 56, Cohort 2: *n* = 57) 50 base pairs (bp) (Cohort 1 *P* = 0.0043, Cohort 2 *P* = 0.29). Visualisation of survival is limited to 72 weeks for comparability between the two cohorts.

Numerical variation of *FLT3*‐ITDs has been associated with outcome in previous reports [10, 30, 33]. We assessed whether individuals with plural ITDs differed in outcome compared to individuals characterised by single ITDs. The median survival of patients with plural *FLT3*‐ITDs was 14.6 months in both C1 and C2 (C1: 24, 95% CI 7.23–NA, C2: 35, 95% CI 8.60–NA), while the median survival of patients with single ITDs was 8.9 months in C1 (68, 95% CI 7.98–15.2) and 20.8 in C2 (88, 95% CI 13.90–45.5), respectively. The differences in median survival were not statistically significant (*P* = 0.24 and *P* = 0.70, respectively) and pointed in opposite directions (Fig. 5A).

High *FLT3*‐ITD t‐VAF was associated with inferior outcome in C2. In C1, patients with t‐VAF < 0.7 (*n* = 84) had a median survival of 13.5 months (95% CI 8.25–24.1) compared to 8.11 (95% CI 6.51–11.9) in patients with t‐VAF ≥ 0.7 (*n* = 27) (*P* = 0.055). Median survival in C2 was 26.6 months (95% CI 14.60–NA) for patients with t‐VAF < 0.7 (*n* = 103) and 7.8 months (95% CI 7.00–17.0) in patients with t‐VAF ≥ 0.7 (*n* = 20) (*P* = 0.00086) (Fig. 5B).

Median survival was significantly shorter in the group characterised by a long ITD sequence (length of LM1 ≥ 50 bp) in C1. Patients with LM1 < 50 bp (*n* = 55) had a median survival of 15.21 months (95% CI 9.2‐NA) as compared to the individuals with LM1 ≥ 50 bp (*n* = 56) who had a median survival of 7.66 months (95% CI 6.93–14–2) (*P* = 0.0043). Median survival in C2 for patients with LM1 < 50 bp (*n* = 66) was 26.6 months (95% CI 13.6–NA) compared to 14.6 months (95% CI 10.3–30) in the group with LM1 ≥ 50 bp (*n* = 57) (*P* = 0.29) (Fig. 5C, Table S7C1,C2).

To identify clinical features associated with prognostic value, we further performed a multivariate cox regression model including age, WBC count, sex, single versus plural *FLT3*‐ITDs, LM1 length and t‐VAF. In C1, the only significant association to poor outcome we identified was female sex, with a hazard ratio of 1.6 compared to males (95% CI: 1.0195–2.609) (*P* = 0.0413). In C2, the same model retained significance for t‐VAF only (*P* = 0.0057) with a hazard ratio of 3.5903 (95% CI: 1.4505–8.887) (Table S8C1,C2).

## 4 Discussion

Here, we have described the distribution of the *FLT3*‐ITD mutated alleles in two separate cohorts of treatment‐naïve AML patients and related it to clinical and molecular characteristics as well as outcome. The associations related to *FLT3*‐ITD mutations, including younger age, female sex, higher WBC counts and higher BM blast percentage as well as cytomorphology, cytogenetics and molecular genetics were mostly consistent with previous reports [11, 14, 15, 18, 52, 53, 54]. Among these observations, the coherent associations between *FLT3*‐ITD status and clinical features nonattributable to downstream effects of the mutation are particularly interesting. This includes the association between *FLT3*‐ITD and female sex and younger age [14, 54] (although not statistically significant in our study). The multivariate cox regression model further identified females as a subgroup that had inferior survival within the *FLT3*‐ITD positive population in C1. Cytogenetic as well as molecular genetic variation in relation to age has previously been described in AML [55, 56], raising questions regarding age‐specific aetiology or whether downstream effects of a mutated gene product may be influenced by age. Such a mechanism has been experimentally substantiated by Porter and colleagues, demonstrating that the phenotypic transition following *FLT3*‐ITD mutations in murine models varied between foetal or neonatal mice and adult mice, where only adult mice developed leukemic phenotypes [57]. Sex‐specific mutational patterns could indicate a similar mechanism. Positive and negative associations between *FLT3*‐ITD mutation status and co‐occurrence or mutual exclusivity with cytogenetic and mutational aberrations like *DNMT3A* and *NPM1* are also recurring findings. Both *DNMT3A* and *NPM1* mutations frequently precede *FLT3*‐ITDs, as inferred by recurring VAF patterns [58] and single cell sequencing data [59, 60]. Experimentally, ectopic *FLT3*‐ITD expression in *FLT3* wild‐type background is known to be detrimental, even in a genetic background that frequently co‐occurs with *FLT3*‐ITDs [61]. In sum, this suggests that the ‘driver’ qualities attributed to *FLT3*‐ITD mutations may at least in part be determined by the gene‐context, including systemic conditions (by the association with age and sex) as well as intracellular gene‐context (as supported by the relationship with cytogenetic and molecular genetic features).

Our results corroborate that intra‐tumour plurality of *FLT3*‐ITD mutations at time of diagnosis is a frequent characteristic of *FLT3*‐ITD mutated AML [10, 15, 20, 29, 30, 32, 33]. Considering the low mutation rate of haematopoietic stem and progenitor cells, estimated to comprise as little as one acquired exonic mutation per decade [62], as well as the relative stability of somatic variants through single AML disease courses [58, 63], it seems implausible that multiple *FLT3*‐ITD mutations are acquired synchronically. Indeed, reports determining *FLT3*‐ITD numerical variation with higher sensitivity assays suggest that plurality of *FLT3*‐ITDs is strongly underestimated [29, 33], which could account for the absence of significant associations observed when comparing patients with one or several *FLT3*‐ITD mutations when assessed by a low sensitivity assay as we have done. A recent report identified up to 16 discrete *FLT3*‐ITD mutations in an individual patient sample, with an average of 3.8 *FLT3*‐ITDs identified per sample when assessed by a deep next‐generation sequencing approach [29]. Longitudinal assessment of *FLT3*‐ITD mutated AML patients [29, 64, 65] as well as single‐cell sequencing studies [59, 60] suggests that discrete *FLT3*‐ITDs are indicative of separate cell populations derived from multiple individual cells that independently acquired *FLT3*‐ITD mutations. Recent work in healthy individuals has demonstrated the ubiquity of mutations known to co‐occur in *FLT3*‐ITD mutated AML, as well as age‐dependent expansion of cell populations characterised by such mutations, including *DNMT3A* and *TET2* [66, 67, 68]. Interpreted along with the high frequency of plural *FLT3*‐ITDs in *FLT3*‐ITD mutated AML, this could suggest that *FLT3*‐ITD mutations are more prevalent than commonly suggested, implying that *FLT3*‐ITD mutations alone are insufficient to trigger AML disease eruption.

As has been well established [11, 20, 69], we demonstrate heterogeneity of *FLT3*‐ITD variant allele distribution at the time of diagnosis. Molecular characteristics of the dominating *FLT3*‐ITD mutation, including length, number of tyrosine residues or insertion integration site, did not correlate with mutational load. However, *FLT3*‐ITD length exceeding 50 bp was associated with inferior overall survival in C1. No clear consensus currently exists regarding the prognostic impact of ITD length, although some studies have suggested that longer ITDs could be detrimental [38, 70]. However, we did not observe a similar association in C2, and the significance of this finding is therefore uncertain. Conversely, high mutational load of *FLT3*‐ITD has been well established to correlate with poor prognosis in AML [12, 13, 19, 71]. This was also evident from our analyses, although only significant in C2. Of note, differences in cohort composition and disparity of treatment, mainly resulting from the temporal separation of the two cohorts, is an important confounder for these results. In C2, *FLT3* mutation status was prospectively determined and used to guide risk‐adapted treatment decisions. This is evident from the significantly higher proportion of *FLT3*‐ITD mutated patients in C2 receiving allogeneic haematopoietic stem cell transplantation (67% vs 24%), which is further reflected in the significantly superior overall survival of *FLT3*‐ITD mutated patients in this cohort. Furthermore, C1 is confined by available sample material and is therefore enriched for patients with high disease burden (i.e. high WBC counts). This could explain the relatively weak impact of t‐VAF on overall survival in this cohort as compared to C2, as WBC count was found to correlate to VAF in both cohorts.

Of note, it was recently reported that risk‐adapted treatment strategies in AML appear to have eliminated the poor risk association with *FLT3*‐ITD [72]. We have confirmed this observation in the HOVON cohorts in a separate study; *FLT3*‐ITD mutated patients in C1 had a significantly worse outcome compared to patients with *FLT3*wt, while there was no difference in overall survival between *FLT3*wt and *FLT3*‐ITD in C2 [73]. Interestingly, in the present study we still identified a poor risk association with high mutational burden within the *FLT3*‐ITD mutated subgroup of this cohort. This relationship between *FLT3*‐ITD VAF and outcome suggests that it is primarily the expansion of *FLT3*‐ITD mutated cell populations rather than the existence of *FLT3*‐ITD mutated cells that correlate with inferior overall survival. Based on the observed co‐existence and often parallel expansion of multiple *FLT3*‐ITD mutations, one cannot exclude that the poor outcome of this subgroup may in part be a function of an underlying systemic mechanism permitting emergence of leukemic properties in multiple *FLT3*‐ITD mutated haematopoietic stem or progenitor cells synchronously. Furthermore, elevated expression of the DNA polymerase terminal deoxynucleotidyl transferase (TdT) is suggested to be related to formation of *FLT3*‐ITDs in AML [74, 75], which may represent a mechanism for the repeated generation of unique length mutations in AML progenitor cells. Such conditions could perhaps account for the complexity of *FLT3*‐ITD mutation distribution and dynamics as well as the persistent finding of *FLT3*‐ITDs as biomarkers of inferior outcome.

## 5 Conclusions

It is clear that *FLT3*‐ITD mutations in AML are characterised by significant inter‐ and intra‐patient heterogeneity. The biological significance of this heterogeneity is not clear, although the poor prognostic impact associated with high VAF and length of the duplicated sequence suggests that these observations are not trivial. It is reasonable to suggest that this heterogeneity could also pose a significant challenge with regards to FLT3‐targeting therapy. Perhaps this could provide a partial explanation for the very limited progress made to date using FLT3‐targeting inhibitors. We suggest that a thorough molecular characterisation of *FLT3*‐ITDs in AML patients undergoing FLT3‐targeting therapy could provide novel biological insight that could ultimately increase predictive and therapeutic precision in *FLT3*‐ITD mutated AML.

## Conflicts of interest

The authors declare no conflict of interest.

## Author contributions

The study was designed by CE and BTG, and the manuscript was written primarily by CE, MH and BTG. CE performed experimental work, data analysis, prepared the data for presentation and interpreted the results. MH contributed to data analyses and interpretation of results, and prepared figures. PJMV provided biological and clinical data and contributed to experimental design. AB, LW, RH and SLB performed experiments and TG and AAH were involved in data analysis. All authors contributed to preparation of the manuscript.

### Peer Review

The peer review history for this article is available at https://publons.com/publon/10.1002/1878‐0261.12961.

## Supporting information

**Fig. S1**. cDNA and gDNA alignment.
**Table S1**. Baseline overview (Cohort 1 versus cohort 2).
**Table S2**. (C1) Cohort 1 (FLT3‐ITD vs no FLT3‐ITD). (C2) Cohort 2 (FLT3‐ITD vs no FLT3‐ITD).
**Table S3**. (C1) Cohort 1 (Single versus plural FLT3‐ITD). (C2) Cohort 2 (Single versus plural FLT3‐ITD).
**Table S4**. (C1) Cohort 1 (FLT3‐ITD t‐VAF </≥ 0.3). (C2) Cohort 2 (FLT3‐ITD t‐VAF </≥ 0.3).
**Table S5**. (C1) Cohort 1 (FLT3‐ITD t‐VAF </≥ 0.7). (C2) Cohort 2 (FLT3‐ITD t‐VAF </≥ 0.7).
**Table S6**. (C1) Cohort 1 (Short versus long FLT3‐ITD major). ST6‐C2 – Cohort 2 (Short versus long FLT3‐ITD major).
**Table S7**. (C1) Survival Analysis Cohort 1 (n = 111). (C2) Survival Analysis Cohort 2 (n = 123).
**Table S8**. Cox Regression Analysis, Cohort 1 (n = 111, number of events=83). (C2) Cox Regression Analysis, Cohort 2 (n = 123, number of events=73).

## Data Availability

Individual participant data from the HOVON1 and HOVON2 cohorts are not publically available. Information about the individual HOVON trials including study protocols is available at http://www.hovon.nl.
