# Supplementary material for: FLT3‐ITD mutations in acute myeloid leukaemia – molecular characteristics, distribution and numerical variation
Source: Mol Oncol. 2021 May 2;15(9):2300–17. doi: 10.1002/1878-0261.12961 (PMC8410560; doi:10.1002/1878-0261.12961)
Supplement: Supplementary file 1 — Fig. S1. cDNA and gDNA alignment. Table S1. Baseline overview (Cohort 1 versus cohort 2). Table S2. (C1) Cohort 1 (FLT3‐ITD vs no FLT3‐ITD). (C2) Cohort 2 (FLT3‐ITD vs no FLT3‐ITD). Table S3. (C1) Cohort 1 (Single versus plural FLT3‐ITD). (C2) Cohort 2 (Single versus plural FLT3‐ITD). Table S4. (C1) Cohort 1 (FLT3‐ITD t‐VAF </≥ 0.3). (C2) Cohort 2 (FLT3‐ITD t‐VAF </≥ 0.3). Table S5. (C1) Cohort 1 (FLT3‐ITD t‐VAF </≥ 0.7). (C2) Cohort 2 (FLT3‐ITD t‐VAF </≥ 0.7). Table S6. (C1) Cohort 1 (Short versus long FLT3‐ITD major). ST6‐C2 – Cohort 2 (Short versus long FLT3‐ITD major). Table S7. (C1) Survival Analysis Cohort 1 (n = 111). (C2) Survival Analysis Cohort 2 (n = 123). Table S8. Cox Regression Analysis, Cohort 1 (n = 111, number of events=83). (C2) Cox Regression Analysis, Cohort 2 (n = 123, number of events=73). [file MOL2-15-2300-s001.docx]

# FLT3-ITD mutations in acute myeloid leukaemia

# – molecular characteristics, distribution and numerical variation

Supplementary material

| **Baseline overview (Cohort 1 versus Cohort 2)** | | | | | |
| --- | --- | --- | --- | --- | --- |
| **ST1** | **Cohort 1** | | **Cohort 2** | | **P-Value** |
| Clinical parameters | |  |  |  |  |
| Age | 46 | (432/432) | 53 | (625/625) | **0.000** |
| PLT | 57 | (431/432) | 59 | (622/625) | 0.161 |
| WBC | 29.3 | (430/432) | 9.8 | (623/625) | **0.000** |
| Female | 215/432 | (49.8%) | 273/625 | (43.7%) | 0.052 |
| Transplantation |  |  |  |  |  |
| Allo-HSCT | 127/432 | (29.4%) | 313/624 | (50.2%) | **0.000** |
| Auto-HSCT | 60/432 | (13.9%) | 61/624 | (9.8%) | **0.049** |
| Predisposition |  |  |  |  |  |
| Prior chrt | 9/432 | (2.1%) | 22/609 | (3.6%) | 0.195 |
| Prior disease | NA |  | 55/624 | (8.8%) | NA |
| Prior ins | NA |  | 3/436 | (0.7%) | NA |
| Prior MDS | 20/432 | (4.6%) | 30/622 | (4.8%) | 1.000 |
| FAB classification |  |  |  |  |  |
| M0 | 18/432 | (4.2%) | 58/604 | (9.6%) | **0.001** |
| M1 | 92/432 | (21.3%) | 143/604 | (23.7%) | 0.408 |
| M2 | 121/432 | (28%) | 202/604 | (33.4%) | 0.066 |
| M4 | 87/432 | (20.1%) | 64/604 | (10.6%) | **0.000** |
| M5 | 107/432 | (24.8%) | 105/604 | (17.4%) | **0.004** |
| M6 | 7/432 | (1.6%) | 28/604 | (4.6%) | **0.008** |
| M7 | NA |  | 4/604 | (0.7%) | NA |
| Karyotype |  |  |  |  |  |
| CBF | 75/432 | (17.4%) | 69/624 | (11.1%) | **0.005** |
| Complex | 28/427 | (6.6%) | 90/622 | (14.5%) | **0.000** |
| inv(16) | 39/432 | (9%) | 28/622 | (4.5%) | **0.004** |
| MK | NA |  | 77/393 | (19.6%) | NA |
| Normal | 193/427 | (45.2%) | 316/625 | (50.6%) | 0.090 |
| + 8 | 34/427 | (8%) | NA |  | NA |
| t(6:9) | 5/427 | (1.2%) | NA |  | NA |
| t(8:21) | 36/432 | (8.3%) | 40/622 | (6.4%) | 0.276 |
| t(9:22) | 5/427 | (1.2%) | NA |  | NA |
| Mutation status |  |  |  |  |  |
| ASXL1 | 20/428 | (4.7%) | 46/501 | (9.2%) | **0.010** |
| CEBPA | 31/431 | (7.2%) | NA |  | NA |
| CEBPA double | 21/432 | (4.9%) | 24/510 | (4.7%) | 1.000 |
| DNMT3A | 93/426 | (21.8%) | 153/501 | (30.5%) | **0.003** |
| FLT3-ITD | 117/432 | (27.1%) | 146/625 | (23.4%) | 0.170 |
| FLT3-TKD | 44/430 | (10.2%) | 42/531 | (7.9%) | 0.214 |
| IDH1 | 31/429 | (7.2%) | 51/501 | (10.2%) | 0.131 |
| IDH2 | 39/429 | (9.1%) | 66/501 | (13.2%) | 0.061 |
| JAK2 | NA |  | 12/501 | (2.4%) | NA |
| KRAS | 4/430 | (0.9%) | NA |  | NA |
| NPM1 | 140/432 | (32.4%) | 186/619 | (30%) | 0.417 |
| NRAS | 41/431 | (9.5%) | NA |  | NA |
| PTPN11 | NA |  | 56/501 | (11.2%) | NA |
| RUNX1 | NA |  | 63/501 | (12.6%) | NA |
| SF3B1 | NA |  | 7/501 | (1.4%) | NA |
| SRSF2 | NA |  | 41/501 | (8.2%) | NA |
| TET2 | NA |  | 57/501 | (11.4%) | NA |
| TP53 | NA |  | 41/501 | (8.2%) | NA |
| WT1 | NA |  | 51/501 | (10.2%) | NA |
|  |  |  |  |  |  |
| PLT: Platelet count (10^9^/L), WBC: White blood cell count (10^9^/L), Allo-HSCT: Allogeneic hematopoietic stem cell transplantation, Auto-HSCT: Autologous hematopoietic stem cell transplantation, chrt: chemotherapy, MDS: Myelodysplastic syndrom, FAB: French-American-British, CBF: Core binding factor leukemia, ASXL1: additional sex combs like 1, CEBPA: CCAAT/enhancer binding protein alpha, DNMT3A: DNA (cytosine-5-)-methyltransferase 3 alpha, FLT3-TKD: fms related tyrosine kinase 3-tyrosine kinase domain mutation, IDH1: isocitrate dehydrogenase (NADP(+)) 1, IDH2: isocitrate dehydrogenase (NADP(+)) 2, KRAS: Kirsten Rat Sarcoma Viral Proto-Oncogene, NPM1: nucleophosmin 1, NRAS: Neuroblastoma RAS Viral Oncogene Homolog, JAK2: Janus kinase 2, PTPN11: Tyrosine-protein phosphatase non-receptor type 11, RUNX1: Runt-related transcription factor 1, SF3B1: Splicing factor 3B subunit 1, SRSF2: Serine And Arginine Rich Splicing Factor 2, TET2: Tet methylcytosine dioxygenase 2, TP53: Tumor protein p53, WT1: Wilms tumor protein | | | | | |

| **Cohort 1 (FLT3-ITD vs no FLT3-ITD)** | | | | | | |  |
| --- | --- | --- | --- | --- | --- | --- | --- |
| **ST2-C1** | **FLT3-ITD** | | **No FLT3-ITD** | | **P-Value** | **FDR adj.** |  |
| Clinical parameters | |  |  |  |  |  |  |
| Age | 47 | (117/117) | 45 | (315/315) | 0.328 | 0.328 | * |
| BM blast % | 81 | (114/117) | 64.5 | (312/315) | 0.000 | **0.000** |  |
| PLT | 64 | (117/117) | 54 | (314/315) | 0.056 | 0.075 |  |
| WBC | 57.8 | (117/117) | 22.1 | (313/315) | 0.000 | **0.000** |  |
| Female | 67/117 | (57.3%) | 148/315 | (47%) | 0.070 |  |  |
| Transplantation |  |  |  |  |  |  |  |
| Allo-HSCT | 29/117 | (24.8%) | 98/315 | (31.1%) | 0.235 |  |  |
| Auto-HSCT | 15/117 | (12.8%) | 45/315 | (14.3%) | 0.756 |  |  |
| Predisposition |  |  |  |  |  |  |  |
| Prior chrt | 0/117 | (0%) | 9/315 | (2.9%) | 0.121 |  |  |
| Prior MDS | 3/117 | (2.6%) | 17/315 | (5.4%) | 0.304 |  |  |
| FAB classification |  |  |  |  |  |  |  |
| M0 | 1/117 | (0.9%) | 17/315 | (5.4%) | 0.053 | 0.080 | * |
| M1 | 34/117 | (29.1%) | 58/315 | (18.4%) | 0.024 | 0.080 |  |
| M2 | 27/117 | (23.1%) | 94/315 | (29.8%) | 0.185 | 0.222 |  |
| M4 | 16/117 | (13.7%) | 71/315 | (22.5%) | 0.043 | 0.080 |  |
| M5 | 38/117 | (32.5%) | 69/315 | (21.9%) | 0.033 | 0.080 |  |
| M6 | 1/117 | (0.9%) | 6/315 | (1.9%) | 0.680 | 0.680 |  |
| Karyotype |  |  |  |  |  |  |  |
| CBF | 3/117 | (2.6%) | 72/315 | (22.9%) | 0.000 | **0.000** | * |
| Complex | 1/116 | (0.9%) | 27/311 | (8.7%) | 0.002 | **0.004** |  |
| inv(16) | 0/117 | (0%) | 39/315 | (12.4%) | 0.000 | **0.000** |  |
| Normal | 85/116 | (73.3%) | 108/311 | (34.7%) | 0.000 | **0.000** |  |
| + 8 | 7/116 | (6%) | 27/311 | (8.7%) | 0.427 | 0.427 |  |
| t(6:9) | 3/116 | (2.6%) | 2/311 | (0.6%) | 0.126 | 0.168 |  |
| t(8:21) | 3/117 | (2.6%) | 33/315 | (10.5%) | 0.006 | **0.010** |  |
| t(9:22) | 0/116 | (0%) | 5/311 | (1.6%) | 0.330 | 0.377 |  |
| Mutation status |  |  |  |  |  |  |  |
| ASXL1 | 1/117 | (0.9%) | 19/311 | (6.1%) | 0.020 | **0.039** | * |
| CEBPA | 7/117 | (6%) | 24/314 | (7.6%) | 0.677 | 0.677 |  |
| CEBPA-double | 3/117 | (2.6%) | 18/315 | (5.7%) | 0.215 | 0.308 |  |
| DNMT3A | 35/117 | (29.9%) | 58/309 | (18.8%) | 0.018 | **0.039** |  |
| FLT3-TKD | 6/117 | (5.1%) | 38/313 | (12.1%) | 0.032 | 0.054 |  |
| IDH1 | 10/117 | (8.5%) | 21/312 | (6.7%) | 0.533 | 0.643 |  |
| IDH2 | 4/117 | (3.4%) | 35/312 | (11.2%) | 0.013 | **0.039** |  |
| KRAS | 0/117 | (0%) | 4/313 | (1.3%) | 0.578 | 0.643 |  |
| NPM1 | 72/117 | (61.5%) | 68/315 | (21.6%) | 0.000 | **0.000** |  |
| NRAS | 0/117 | (0%) | 41/314 | (13.1%) | 0.000 | **0.000** |  |
|  |  |  |  |  |  |  |  |
| BM blast %: Bone marrow blast percentage, PLT: Platelet count (10^9^/L), WBC: White blood cell count (10^9^/L), Allo-HSCT: Allogeneic hematopoietic stem cell transplantation, Auto-HSCT: Autologous hematopoietic stem cell transplantation, chrt: chemotherapy, MDS: Myelodysplastic syndrom, FAB: French-American-British, CBF: Core binding factor leukemia, ASXL1: additional sex combs like 1, CEBPA: CCAAT/enhancer binding protein alpha, DNMT3A: DNA (cytosine-5-)-methyltransferase 3 alpha, FLT3-TKD: fms related tyrosine kinase 3-tyrosine kinase domain mutation, IDH1: isocitrate dehydrogenase (NADP(+)) 1, IDH2: isocitrate dehydrogenase (NADP(+)) 2, KRAS: Kirsten Rat Sarcoma Viral Proto-Oncogene, NPM1: nucleophosmin 1, NRAS: Neuroblastoma RAS Viral Oncogene Homolog. * indicates groups of p-values of which the false discovery rate (fdr) is calulated. | | | | | | |  |

| **Cohort 2 (FLT3-ITD vs no FLT3-ITD)** | | | | | | |  |
| --- | --- | --- | --- | --- | --- | --- | --- |
| **ST2-C2** | **FLT3-ITD** | | **No FLT3-ITD** | | **P-Value** | **FDR adj.** |  |
| Clinical parameters | |  |  |  |  |  |  |
| Age | 50 | (146/146) | 55 | (479/479) | 0.042 | 0.062 | * |
| PLT | 60 | (145/146) | 58 | (477/479) | 0.608 | 0.608 |  |
| WBC | 37.7 | (146/146) | 6.2 | (477/479) | 0.000 | **0.000** |  |
| Female | 74/146 | (50.7%) | 199/479 | (41.5%) | 0.057 |  |  |
| Transplantation |  |  |  |  |  |  |  |
| Allo-HSCT | 94/146 | (64.4%) | 219/478 | (45.8%) | 0.000 |  |  |
| Auto-HSCT | 15/146 | (10.3%) | 46/478 | (9.6%) | 0.874 |  |  |
| Predisposition |  |  |  |  |  |  |  |
| Prior chrt | 6/142 | (4.2%) | 16/467 | (3.4%) | 0.614 |  |  |
| Prior disease | 8/146 | (5.5%) | 47/478 | (9.8%) | 0.132 |  |  |
| Prior ins | 0/97 | (0%) | 3/339 | (0.9%) | 1.000 |  |  |
| Prior MDS | 7/145 | (4.8%) | 23/477 | (4.8%) | 1.000 |  |  |
| FAB classification |  |  |  |  |  |  |  |
| M0 | 6/140 | (4.3%) | 52/464 | (11.2%) | 0.014 | **0.048** | * |
| M1 | 48/140 | (34.3%) | 95/464 | (20.5%) | 0.001 | **0.010** |  |
| M2 | 42/140 | (30%) | 160/464 | (34.5%) | 0.358 | 0.418 |  |
| M4 | 8/140 | (5.7%) | 56/464 | (12.1%) | 0.040 | 0.094 |  |
| M5 | 32/140 | (22.9%) | 73/464 | (15.7%) | 0.057 | 0.099 |  |
| M6 | 3/140 | (2.1%) | 25/464 | (5.4%) | 0.166 | 0.232 |  |
| M7 | 1/140 | (0.7%) | 3/464 | (0.6%) | 1.000 | 1.000 |  |
| Karyotype |  |  |  |  |  |  |  |
| CBF | 7/146 | (4.8%) | 62/478 | (13%) | 0.004 | **0.006** | * |
| Complex | 1/146 | (0.7%) | 89/476 | (18.7%) | 0.000 | **0.000** |  |
| inv(16) | 2/146 | (1.4%) | 26/476 | (5.5%) | 0.039 | **0.047** |  |
| MK | 0/108 | (0%) | 77/285 | (27%) | 0.000 | **0.000** |  |
| Normal | 108/146 | (74%) | 208/479 | (43.4%) | 0.000 | **0.000** |  |
| t(8:21) | 5/146 | (3.4%) | 35/476 | (7.4%) | 0.121 | 0.121 |  |
| Mutation status |  |  |  |  |  |  |  |
| ASXL1 | 3/128 | (2.3%) | 43/373 | (11.5%) | 0.001 | **0.004** | * |
| CEBPA double | 4/121 | (3.3%) | 20/389 | (5.1%) | 0.622 | 0.778 |  |
| DNMT3A | 55/128 | (43%) | 98/373 | (26.3%) | 0.001 | **0.003** |  |
| FLT3-TKD | 11/130 | (8.5%) | 31/401 | (7.7%) | 0.852 | 0.913 |  |
| IDH1 | 13/128 | (10.2%) | 38/373 | (10.2%) | 1.000 | 1.000 |  |
| IDH2 | 21/128 | (16.4%) | 45/373 | (12.1%) | 0.226 | 0.377 |  |
| JAK2 | 0/128 | (0%) | 12/373 | (3.2%) | 0.043 | 0.091 |  |
| NPM1 | 79/146 | (54.1%) | 107/473 | (22.6%) | 0.000 | **0.000** |  |
| PTPN11 | 9/128 | (7%) | 47/373 | (12.6%) | 0.103 | 0.194 |  |
| RUNX1 | 13/128 | (10.2%) | 50/373 | (13.4%) | 0.440 | 0.600 |  |
| SF3B1 | 3/128 | (2.3%) | 4/373 | (1.1%) | 0.379 | 0.569 |  |
| SRSF2 | 5/128 | (3.9%) | 36/373 | (9.7%) | 0.041 | 0.091 |  |
| TET2 | 13/128 | (10.2%) | 44/373 | (11.8%) | 0.747 | 0.862 |  |
| TP53 | 1/128 | (0.8%) | 40/373 | (10.7%) | 0.000 | **0.001** |  |
| WT1 | 20/128 | (15.6%) | 31/373 | (8.3%) | 0.027 | 0.080 |  |
|  |  |  |  |  |  |  |  |
| PLT: Platelet count (10^9^/L), WBC: White blood cell count (10^9^/L), Allo-HSCT: Allogeneic hematopoietic stem cell transplantation, Auto-HSCT: Autologous hematopoietic stem cell transplantation, chrt: chemotherapy, ins: insecticide, MDS: Myelodysplastic syndrom, FAB: French-American-British, CBF: Core binding factor leukemia, MK: monosomal karyotype, ASXL1: additional sex combs like 1, CEBPA: CCAAT/enhancer binding protein alpha, DNMT3A: DNA (cytosine-5-)-methyltransferase 3 alpha, FLT3-TKD: fms related tyrosine kinase 3-tyrosine kinase domain mutation, IDH1: isocitrate dehydrogenase (NADP(+)) 1, IDH2: isocitrate dehydrogenase (NADP(+)) 2, KRAS: Kirsten Rat Sarcoma Viral Proto-Oncogene, NPM1: nucleophosmin 1, NRAS: Neuroblastoma RAS Viral Oncogene Homolog, JAK2: Janus kinase 2, PTPN11: Tyrosine-protein phosphatase non-receptor type 11, RUNX1: Runt-related transcription factor 1, SF3B1: Splicing factor 3B subunit 1, SRSF2: Serine And Arginine Rich Splicing Factor 2, TET2: Tet methylcytosine dioxygenase 2, TP53: Tumor protein p53, WT1: Wilms tumor protein.* indicates groups of p-values of which the false discovery rate (fdr) is calulated. | | | | | | |  |


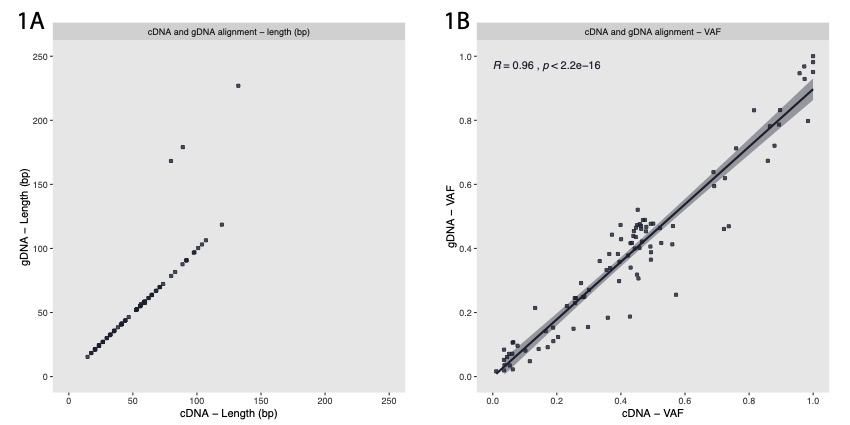


**Supplementary Figure 1 – cDNA and gDNA alignment:** A. Correlation between estimated fragment length in base pairs (bp) in complementary DNA (cDNA) and genomic DNA (gDNA). The gDNA sequence of three samples include a 90bp intron, accounting for the discrepancy in the gDNA/cDNA ratio of the three samples with the longest ITDs. B. Correlation between cDNA and gDNA estimated variant allele frequency (VAF). n=95, R=0.96, p>2.2e-16. Strength of linear associations was assessed by Pearson correlation coeffecient. The shaded area indicates the 95% confidence interval.

| **Cohort 1 (Single versus plural FLT3-ITD)** | | | | | | |  |
| --- | --- | --- | --- | --- | --- | --- | --- |
| **ST3-C1** | **Single** | | **Plural** | | **P-Value** | **FDR adj.** |  |
| Clinical parameters | |  |  |  |  |  |  |
| Age | 47 | (87/87) | 42.5 | (24/24) | 0.450 | 0.599 | * |
| BM blast % | 82 | (84/87) | 73 | (24/24) | 0.359 | 0.599 |  |
| PLT | 64 | (87/87) | 59.5 | (24/24) | 0.270 | 0.599 |  |
| WBC | 60 | (87/87) | 57.9 | (24/24) | 0.977 | 0.977 |  |
| Female | 54/87 | (62.1%) | 11/24 | (45.8%) | 0.168 |  |  |
| Transplantation |  |  |  |  |  |  |  |
| Allo-HSCT | 21/87 | (24.1%) | 6/24 | (25%) | 1.000 |  |  |
| Auto-HSCT | 12/87 | (13.8%) | 3/24 | (12.5%) | 1.000 |  |  |
| Predisposition |  |  |  |  |  |  |  |
| Prior chrt | 0/87 | (0%) | 0/24 | (0%) | 1.000 |  |  |
| Prior MDS | 2/87 | (2.3%) | 0/24 | (0%) | 1.000 |  |  |
| FAB classification |  |  |  |  |  |  |  |
| M0 | 1/87 | (1.1%) | 0/24 | (0%) | 1.000 | 1.000 | * |
| M1 | 26/87 | (29.9%) | 6/24 | (25%) | 0.800 | 1.000 |  |
| M2 | 20/87 | (23%) | 6/24 | (25%) | 0.792 | 1.000 |  |
| M4 | 13/87 | (14.9%) | 2/24 | (8.3%) | 0.517 | 1.000 |  |
| M5 | 27/87 | (31%) | 10/24 | (41.7%) | 0.338 | 1.000 |  |
| M6 | 0/87 | (0%) | 0/24 | (0%) | 1.000 | 1.000 |  |
| Karyotype |  |  |  |  |  |  |  |
| CBF | 2/87 | (2.3%) | 1/24 | (4.2%) | 0.522 | 1.000 | * |
| Complex | 1/86 | (1.2%) | 0/24 | (0%) | 1.000 | 1.000 |  |
| inv(16) | 0/87 | (0%) | 0/24 | (0%) | 1.000 | 1.000 |  |
| Normal | 62/86 | (72.1%) | 19/24 | (79.2%) | 0.605 | 1.000 |  |
| + 8 | 5/86 | (5.8%) | 2/24 | (8.3%) | 0.645 | 1.000 |  |
| t(6:9) | 3/86 | (3.5%) | 0/24 | (0%) | 1.000 | 1.000 |  |
| t(8:21) | 2/87 | (2.3%) | 1/24 | (4.2%) | 0.522 | 1.000 |  |
| t(9:22) | 0/86 | (0%) | 0/24 | (0%) | 1.000 | 1.000 |  |
| Mutation status |  |  |  |  |  |  |  |
| ASXL1 | 1/87 | (1.1%) | 0/24 | (0%) | 1.000 | 1.000 | * |
| CEBPA | 6/87 | (6.9%) | 1/24 | (4.2%) | 1.000 | 1.000 |  |
| CEBPA-double | 3/87 | (3.4%) | 0/24 | (0%) | 1.000 | 1.000 |  |
| DNMT3A | 27/87 | (31%) | 8/24 | (33.3%) | 0.809 | 1.000 |  |
| FLT3-TKD | 3/87 | (3.4%) | 3/24 | (12.5%) | 0.114 | 1.000 |  |
| IDH1 | 7/87 | (8%) | 2/24 | (8.3%) | 1.000 | 1.000 |  |
| IDH2 | 2/87 | (2.3%) | 2/24 | (8.3%) | 0.204 | 1.000 |  |
| KRAS | 0/87 | (0%) | 0/24 | (0%) | 1.000 | 1.000 |  |
| NPM1 | 52/87 | (59.8%) | 17/24 | (70.8%) | 0.354 | 1.000 |  |
| NRAS | 0/87 | (0%) | 0/24 | (0%) | 1.000 | 1.000 |  |
|  |  |  |  |  |  |  |  |
| BM blast %: Bone marrow blast percentage, PLT: Platelet count (10^9^/L), WBC: White blood cell count (10^9^/L), Allo-HSCT: Allogeneic hematopoietic stem cell transplantation, Auto-HSCT: Autologous hematopoietic stem cell transplantation, chrt: chemotherapy, MDS: Myelodysplastic syndrom, FAB: French-American-British, CBF: Core binding factor leukemia, ASXL1: additional sex combs like 1, CEBPA: CCAAT/enhancer binding protein alpha, DNMT3A: DNA (cytosine-5-)-methyltransferase 3 alpha, FLT3-TKD: fms related tyrosine kinase 3-tyrosine kinase domain mutation, IDH1: isocitrate dehydrogenase (NADP(+)) 1, IDH2: isocitrate dehydrogenase (NADP(+)) 2, KRAS: Kirsten Rat Sarcoma Viral Proto-Oncogene, NPM1: nucleophosmin 1, NRAS: Neuroblastoma RAS Viral Oncogene Homolog. * indicates groups of p-values of which the false discovery rate (fdr) is calulated. | | | | | | |  |

| **Cohort 2 (Single versus plural FLT3-ITD)** | | | | | | |  |
| --- | --- | --- | --- | --- | --- | --- | --- |
| **ST3-C2** | **Single** | | **Plural** | | **P-Value** | **FDR adj.** |  |
| **Clinical parameters** | |  |  |  |  |  |  |
| Age | 49.5 | (88/88) | 53 | (35/35) | 0.682 | 0.773 | * |
| PLT | 64 | (88/88) | 70 | (35/35) | 0.773 | 0.773 |  |
| WBC | 41.7 | (88/88) | 46.9 | (35/35) | 0.558 | 0.773 |  |
| Female | 41/88 | (46.6%) | 19/35 | (54.3%) | 1.000 |  |  |
| Transplantation |  |  |  |  |  |  |  |
| Allo-HSCT | 58/88 | (65.9%) | 24/35 | (68.6%) | 0.835 |  |  |
| Auto-HSCT | 10/88 | (11.4%) | 3/35 | (8.6%) | 0.756 |  |  |
| Predisposition |  |  |  |  |  |  |  |
| Prior chrt | 5/85 | (5.9%) | 0/34 | (0%) | 0.320 |  |  |
| Prior disease | 7/88 | (8%) | 0/35 | (0%) | 0.190 |  |  |
| Prior ins | 0/55 | (0%) | 0/29 | (0%) | 1.000 |  |  |
| Prior MDS | 6/87 | (6.9%) | 0/35 | (0%) | 0.181 |  |  |
| FAB classification |  |  |  |  |  |  |  |
| M0 | 3/84 | (3.6%) | 3/35 | (8.6%) | 0.358 | 0.970 | * |
| M1 | 26/84 | (31%) | 13/35 | (37.1%) | 0.527 | 0.970 |  |
| M2 | 25/84 | (29.8%) | 11/35 | (31.4%) | 1.000 | 1.000 |  |
| M4 | 6/84 | (7.1%) | 2/35 | (5.7%) | 1.000 | 1.000 |  |
| M5 | 20/84 | (23.8%) | 6/35 | (17.1%) | 0.476 | 0.970 |  |
| M6 | 3/84 | (3.6%) | 0/35 | (0%) | 0.554 | 0.970 |  |
| M7 | 1/84 | (1.2%) | 0/35 | (0%) | 1.000 | 1.000 |  |
| Karyotype |  |  |  |  |  |  |  |
| CBF | 4/88 | (4.5%) | 0/35 | (0%) | 0.577 | 0.721 | * |
| Complex | 0/88 | (0%) | 1/35 | (2.9%) | 0.285 | 0.721 |  |
| inv(16) | 1/88 | (1.1%) | 0/35 | (0%) | 1.000 | 1.000 |  |
| Normal | 67/88 | (76.1%) | 24/35 | (68.6%) | 0.495 | 0.721 |  |
| t(8:21) | 3/88 | (3.4%) | 0/35 | (0%) | 0.557 | 0.721 |  |
| Mutation status |  |  |  |  |  |  |  |
| ASXL1 | 3/85 | (3.5%) | 0/32 | (0%) | 0.561 | 1.000 | * |
| CEBPA double | 3/74 | (4.1%) | 1/27 | (3.7%) | 1.000 | 1.000 |  |
| DNMT3A | 41/85 | (48.2%) | 13/32 | (40.6%) | 0.535 | 1.000 |  |
| FLT3-TKD | 7/86 | (8.1%) | 4/32 | (12.5%) | 0.487 | 1.000 |  |
| IDH1 | 9/85 | (10.6%) | 2/32 | (6.3%) | 0.725 | 1.000 |  |
| IDH2 | 16/85 | (18.8%) | 5/32 | (15.6%) | 0.792 | 1.000 |  |
| JAK2 | 0/85 | (0%) | 0/32 | (0%) | 1.000 | 1.000 |  |
| NPM1 | 48/88 | (54.5%) | 19/35 | (54.3%) | 1.000 | 1.000 |  |
| PTPN11 | 7/85 | (8.2%) | 1/32 | (3.1%) | 0.443 | 1.000 |  |
| RUNX1 | 9/85 | (10.6%) | 4/32 | (12.5%) | 0.749 | 1.000 |  |
| SF3B1 | 2/85 | (2.4%) | 1/32 | (3.1%) | 1.000 | 1.000 |  |
| SRSF2 | 3/85 | (3.5%) | 1/32 | (3.1%) | 1.000 | 1.000 |  |
| TET2 | 11/85 | (12.9%) | 1/32 | (3.1%) | 0.176 | 1.000 |  |
| TP53 | 1/85 | (1.2%) | 0/32 | (0%) | 1.000 | 1.000 |  |
| WT1 | 13/85 | (15.3%) | 6/32 | (18.8%) | 0.779 | 1.000 |  |
|  |  |  |  |  |  |  |  |
| PLT: Platelet count (10^9^/L), WBC: White blood cell count (10^9^/L), Allo-HSCT: Allogeneic hematopoietic stem cell transplantation, Auto-HSCT: Autologous hematopoietic stem cell transplantation, chrt: chemotherapy, ins: insecticide, MDS: Myelodysplastic syndrom, FAB: French-American-British, CBF: Core binding factor leukemia, ASXL1: additional sex combs like 1, CEBPA: CCAAT/enhancer binding protein alpha, DNMT3A: DNA (cytosine-5-)-methyltransferase 3 alpha, FLT3-TKD: fms related tyrosine kinase 3-tyrosine kinase domain mutation, IDH1: isocitrate dehydrogenase (NADP(+)) 1, IDH2: isocitrate dehydrogenase (NADP(+)) 2, KRAS: Kirsten Rat Sarcoma Viral Proto-Oncogene, NPM1: nucleophosmin 1, NRAS: Neuroblastoma RAS Viral Oncogene Homolog, JAK2: Janus kinase 2, PTPN11: Tyrosine-protein phosphatase non-receptor type 11, RUNX1: Runt-related transcription factor 1, SF3B1: Splicing factor 3B subunit 1, SRSF2: Serine And Arginine Rich Splicing Factor 2, TET2: Tet methylcytosine dioxygenase 2, TP53: Tumor protein p53, WT1: Wilms tumor protein. * indicates groups of p-values of which the false discovery rate (fdr) is calulated. | | | | | | |  |

| **Cohort 1 (FLT3-ITD t-VAF </≥ 0.3)** | | | | | | |  |
| --- | --- | --- | --- | --- | --- | --- | --- |
| **ST4-C1** | **<0.3** | | **≥0.3** | | **P-Value** | **FDR adj.** |  |
| Clinical parameters | |  |  |  |  |  |  |
| Age | 54 | 17/17 | 46 | 94/94 | 0.246 | 0.493 | * |
| BM blast % | 78.5 | 16/17 | 81.5 | 92/94 | 0.528 | 0.644 |  |
| PLT | 70 | 17/17 | 57 | 94/94 | 0.644 | 0.644 |  |
| WBC | 23 | 17/17 | 68.8 | 94/94 | 0.002 | **0.009** |  |
| Female | 12/17 | (70.6%) | 53/94 | (53/94%) | 0.301 |  |  |
| Transplantation |  |  |  |  |  |  |  |
| Allo-HSCT | 7/17 | (41.2%) | 20/94 | (21.3%) | 0.121 |  |  |
| Auto-HSCT | 3/17 | (17.6%) | 12/94 | (12.8%) | 0.699 |  |  |
| Predisposition |  |  |  |  |  |  |  |
| Prior chrt | 0/17 | (0%) | 0/94 | (0%) | 1.000 |  |  |
| Prior MDS | 1/17 | (5.9%) | 1/94 | (1.1%) | 0.284 |  |  |
| FAB classification |  |  |  |  |  |  |  |
| M0 | 0/17 | (0%) | 1/94 | (1.1%) | 1.000 | 1.000 | * |
| M1 | 7/17 | (41.2%) | 25/94 | (26.6%) | 0.251 | 0.753 |  |
| M2 | 5/17 | (29.4%) | 21/94 | (22.3%) | 0.541 | 1.000 |  |
| M4 | 2/17 | (11.8%) | 13/94 | (13.8%) | 1.000 | 1.000 |  |
| M5 | 3/17 | (17.6%) | 34/94 | (36.2%) | 0.169 | 0.753 |  |
| M6 | 0/17 | (0%) | 0/94 | (0%) | 1.000 | 1.000 |  |
| Karyotype |  |  |  |  |  |  |  |
| CBF | 1/17 | (5.9%) | 2/94 | (2.1%) | 0.396 | 0.633 | * |
| Complex | 1/17 | (5.9%) | 0/93 | (0%) | 0.155 | 0.633 |  |
| inv(16) | 0/17 | (0%) | 0/94 | (0%) | 1.000 | 1.000 |  |
| Normal | 11/17 | (64.7%) | 70/93 | (75.3%) | 0.379 | 0.633 |  |
| + 8 | 2/17 | (11.8%) | 5/93 | (5.4%) | 0.295 | 0.633 |  |
| t(6:9) | 0/17 | (0%) | 3/93 | (3.2%) | 1.000 | 1.000 |  |
| t(8:21) | 1/17 | (5.9%) | 2/94 | (2.1%) | 0.396 | 0.633 |  |
| t(9:22) | 0/17 | (0%) | 0/93 | (0%) | 1.000 | 1.000 |  |
| Mutation status |  |  |  |  |  |  |  |
| ASXL1 | 1/17 | (5.9%) | 0/94 | (0%) | 0.153 | 0.255 | * |
| CEBPA | 3/17 | (17.6%) | 4/94 | (4.3%) | 0.071 | 0.178 |  |
| CEBPA-double | 3/17 | (17.6%) | 0/94 | (0%) | 0.003 | **0.031** |  |
| DNMT3A | 4/17 | (23.5%) | 31/94 | (33%) | 0.575 | 0.719 |  |
| FLT3-TKD | 3/17 | (17.6%) | 3/94 | (3.2%) | 0.045 | 0.150 |  |
| IDH1 | 3/17 | (17.6%) | 6/94 | (6.4%) | 0.140 | 0.255 |  |
| IDH2 | 3/17 | (17.6%) | 1/94 | (1.1%) | 0.011 | 0.055 |  |
| KRAS | 0/17 | (0%) | 0/94 | (0%) | 1.000 | 1.000 |  |
| NPM1 | 8/17 | (47.1%) | 61/94 | (64.9%) | 0.183 | 0.261 |  |
| NRAS | 0/17 | (0%) | 0/94 | (0%) | 1.000 | 1.000 |  |
|  |  |  |  |  |  |  |  |
| BM blast %: Bone marrow blast percentage, PLT: Platelet count (10^9^/L), WBC: White blood cell count (10^9^/L), Allo-HSCT: Allogeneic hematopoietic stem cell transplantation, Auto-HSCT: Autologous hematopoietic stem cell transplantation, chrt: chemotherapy, MDS: Myelodysplastic syndrom, FAB: French-American-British, CBF: Core binding factor leukemia, ASXL1: additional sex combs like 1, CEBPA: CCAAT/enhancer binding protein alpha, DNMT3A: DNA (cytosine-5-)-methyltransferase 3 alpha, FLT3-TKD: fms related tyrosine kinase 3-tyrosine kinase domain mutation, IDH1: isocitrate dehydrogenase (NADP(+)) 1, IDH2: isocitrate dehydrogenase (NADP(+)) 2, KRAS: Kirsten Rat Sarcoma Viral Proto-Oncogene, NPM1: nucleophosmin 1, NRAS: Neuroblastoma RAS Viral Oncogene Homolog. * indicates groups of p-values of which the false discovery rate (fdr) is calulated. | | | | | | |  |

| **Cohort 2 (FLT3-ITD t-VAF </≥ 0.3)** | | | | | | |  |
| --- | --- | --- | --- | --- | --- | --- | --- |
| **ST4-C2** | **<0.3** | | **≥0.3** | | **P-Value** | **FDR adj.** |  |
| Clinical parameters | |  |  |  |  |  |  |
| Age | 49 | (39/39) | 52.5 | (84/84) | 0.879 | 0.879 | * |
| PLT | 60 | (39/39) | 70 | (84/84) | 0.223 | 0.335 |  |
| WBC | 19.5 | (39/39) | 51.9 | (84/84) | 0.005 | **0.016** |  |
| Female | 19/39 | (48.7%) | 47/84 | (56%) | 0.560 |  |  |
| Transplantation |  |  |  |  |  |  |  |
| Allo-HSCT | 29/39 | (74.4%) | 53/84 | (63.1%) | 0.304 |  |  |
| Auto-HSCT | 3/39 | (7.7%) | 10/84 | (11.9%) | 0.753 |  |  |
| Predisposition |  |  |  |  |  |  |  |
| Prior chrt | 2/38 | (5.3%) | 3/81 | (3.7%) | 0.654 |  |  |
| Prior disease | 1/39 | (2.6%) | 6/84 | (7.1%) | 0.430 |  |  |
| Prior ins | 0/26 | (0%) | 0/58 | (0%) | 1.000 |  |  |
| Prior MDS | 0/38 | (0%) | 6/84 | (7.1%) | 0.175 |  |  |
| FAB classification |  |  |  |  |  |  |  |
| M0 | 3/39 | (7.7%) | 3/80 | (3.8%) | 0.392 | 0.750 | * |
| M1 | 11/39 | (28.2%) | 28/80 | (35%) | 0.535 | 0.750 |  |
| M2 | 10/39 | (25.6%) | 26/80 | (32.5%) | 0.526 | 0.750 |  |
| M4 | 2/39 | (5.1%) | 6/80 | (7.5%) | 1.000 | 1.000 |  |
| M5 | 10/39 | (25.6%) | 16/80 | (20%) | 0.488 | 0.750 |  |
| M6 | 3/39 | (7.7%) | 0/80 | (0%) | 0.033 | 0.234 |  |
| M7 | 0/39 | (0%) | 1/80 | (1.3%) | 1.000 | 1.000 |  |
| Karyotype |  |  |  |  |  |  |  |
| CBF | 3/39 | (7.7%) | 1/84 | (1.2%) | 0.094 | 0.470 | * |
| Complex | 0/39 | (0%) | 1/84 | (1.2%) | 1.000 | 1.000 |  |
| inv(16) | 1/39 | (2.6%) | 0/84 | (0%) | 0.317 | 0.528 |  |
| Normal | 27/39 | (69.2%) | 64/84 | (76.2%) | 0.508 | 0.635 |  |
| t(8:21) | 2/39 | (5.1%) | 1/84 | (1.2%) | 0.236 | 0.528 |  |
| Mutation status |  |  |  |  |  |  |  |
| ASXL1 | 2/35 | (5.7%) | 1/82 | (1.2%) | 0.213 | 0.525 | * |
| CEBPA double | 2/33 | (6.1%) | 2/68 | (2.9%) | 0.595 | 0.892 |  |
| DNMT3A | 8/35 | (22.9%) | 46/82 | (56.1%) | 0.001 | **0.017** |  |
| FLT3-TKD | 7/36 | (19.4%) | 4/82 | (4.9%) | 0.033 | 0.248 |  |
| IDH1 | 4/35 | (11.4%) | 7/82 | (8.5%) | 0.731 | 0.940 |  |
| IDH2 | 9/35 | (25.7%) | 12/82 | (14.6%) | 0.190 | 0.525 |  |
| JAK2 | 0/35 | (0%) | 0/82 | (0%) | 1.000 | 1.000 |  |
| NPM1 | 18/39 | (46.2%) | 49/84 | (58.3%) | 0.245 | 0.525 |  |
| PTPN11 | 5/35 | (14.3%) | 3/82 | (3.7%) | 0.051 | 0.255 |  |
| RUNX1 | 3/35 | (8.6%) | 10/82 | (12.2%) | 0.752 | 0.940 |  |
| SF3B1 | 1/35 | (2.9%) | 2/82 | (2.4%) | 1.000 | 1.000 |  |
| SRSF2 | 2/35 | (5.7%) | 2/82 | (2.4%) | 0.582 | 0.892 |  |
| TET2 | 6/35 | (17.1%) | 6/82 | (7.3%) | 0.179 | 0.525 |  |
| TP53 | 1/35 | (2.9%) | 0/82 | (0%) | 0.299 | 0.561 |  |
| WT1 | 6/35 | (17.1%) | 13/82 | (15.9%) | 1.000 | 1.000 |  |
|  |  |  |  |  |  |  |  |
| PLT: Platelet count (10^9^/L), WBC: White blood cell count (10^9^/L), Allo-HSCT: Allogeneic hematopoietic stem cell transplantation, Auto-HSCT: Autologous hematopoietic stem cell transplantation, chrt: chemotherapy, ins: insecticide, MDS: Myelodysplastic syndrom, FAB: French-American-British, CBF: Core binding factor leukemia, ASXL1: additional sex combs like 1, CEBPA: CCAAT/enhancer binding protein alpha, DNMT3A: DNA (cytosine-5-)-methyltransferase 3 alpha, FLT3-TKD: fms related tyrosine kinase 3-tyrosine kinase domain mutation, IDH1: isocitrate dehydrogenase (NADP(+)) 1, IDH2: isocitrate dehydrogenase (NADP(+)) 2, KRAS: Kirsten Rat Sarcoma Viral Proto-Oncogene, NPM1: nucleophosmin 1, NRAS: Neuroblastoma RAS Viral Oncogene Homolog, JAK2: Janus kinase 2, PTPN11: Tyrosine-protein phosphatase non-receptor type 11, RUNX1: Runt-related transcription factor 1, SF3B1: Splicing factor 3B subunit 1, SRSF2: Serine And Arginine Rich Splicing Factor 2, TET2: Tet methylcytosine dioxygenase 2, TP53: Tumor protein p53, WT1: Wilms tumor protein. * indicates groups of p-values of which the false discovery rate (fdr) is calulated. | | | | | | |  |

| **Cohort 1 (FLT3-ITD t-VAF </≥ 0.7)** | | | | | | |  |
| --- | --- | --- | --- | --- | --- | --- | --- |
| **ST5-C1** | **<0.7** | | **≥0.7** | | **P-Value** | **FDR adj.** |  |
| Clinical parameters | |  |  |  |  |  |  |
| Age | 44 | (84/84) | 47 | (27/27) | 0.997 | 0.997 | * |
| BM blast % | 79 | (81/84) | 83 | (27/27) | 0.597 | 0.796 |  |
| PLT | 66.5 | (84/84) | 55 | (27/27) | 0.423 | 0.796 |  |
| WBC | 45 | (84/84) | 115.3 | (27/27) | 0.001 | **0.004** |  |
| Female | 50/84 | (59.5%) | 15/27 | (55.6%) | 0.823 |  |  |
| Transplantation |  |  |  |  |  |  |  |
| Allo-HSCT | 20/84 | (23.8%) | 7/27 | (25.9%) | 0.802 |  |  |
| Auto-HSCT | 14/84 | (16.7%) | 1/27 | (3.7%) | 0.111 |  |  |
| Predisposition |  |  |  |  |  |  |  |
| Prior chrt | 0/84 | (0%) | 0/27 | (0%) | 1.000 |  |  |
| Prior MDS | 2/84 | (2.4%) | 0/27 | (0%) | 1.000 |  |  |
| FAB classification |  |  |  |  |  |  |  |
| M0 | 0/84 | (0%) | 1/27 | (3.7%) | 0.243 | 0.716 | * |
| M1 | 28/84 | (33.3%) | 4/27 | (14.8%) | 0.087 | 0.522 |  |
| M2 | 19/84 | (22.6%) | 7/27 | (25.9%) | 0.795 | 0.954 |  |
| M4 | 11/84 | (13.1%) | 4/27 | (14.8%) | 0.757 | 0.954 |  |
| M5 | 26/84 | (31%) | 11/27 | (40.7%) | 0.358 | 0.716 |  |
| M6 | 0/84 | (0%) | 0/27 | (0%) | 1.000 | 1.000 |  |
| Karyotype |  |  |  |  |  |  |  |
| CBF | 3/84 | (3.6%) | 0/27 | (0%) | 1.000 | 1.000 | * |
| Complex | 1/84 | (1.2%) | 0/26 | (0%) | 1.000 | 1.000 |  |
| inv(16) | 0/84 | (0%) | 0/27 | (0%) | 1.000 | 1.000 |  |
| Normal | 61/84 | (72.6%) | 20/26 | (76.9%) | 0.801 | 1.000 |  |
| + 8 | 6/84 | (7.1%) | 1/26 | (3.8%) | 1.000 | 1.000 |  |
| t(6:9) | 2/84 | (2.4%) | 1/26 | (3.8%) | 0.559 | 1.000 |  |
| t(8:21) | 3/84 | (3.6%) | 0/27 | (0%) | 1.000 | 1.000 |  |
| t(9:22) | 0/84 | (0%) | 0/26 | (0%) | 1.000 | 1.000 |  |
| Mutation status |  |  |  |  |  |  |  |
| ASXL1 | 1/84 | (1.2%) | 0/27 | (0%) | 1.000 | 1.000 | * |
| CEBPA | 7/84 | (8.3%) | 0/27 | (0%) | 0.192 | 0.958 |  |
| CEBPA-double | 3/84 | (3.6%) | 0/27 | (0%) | 1.000 | 1.000 |  |
| DNMT3A | 25/84 | (29.8%) | 10/27 | (37%) | 0.485 | 1.000 |  |
| FLT3-TKD | 4/84 | (4.8%) | 2/27 | (7.4%) | 0.632 | 1.000 |  |
| IDH1 | 9/84 | (10.7%) | 0/27 | (0%) | 0.110 | 0.958 |  |
| IDH2 | 4/84 | (4.8%) | 0/27 | (0%) | 0.570 | 1.000 |  |
| KRAS | 0/84 | (0%) | 0/27 | (0%) | 1.000 | 1.000 |  |
| NPM1 | 50/84 | (59.5%) | 19/27 | (70.4%) | 0.367 | 1.000 |  |
| NRAS | 0/84 | (0%) | 0/27 | (0%) | 1.000 | 1.000 |  |
|  |  |  |  |  |  |  |  |
| BM blast %: Bone marrow blast percentage, PLT: Platelet count (10^9^/L), WBC: White blood cell count (10^9^/L), Allo-HSCT: Allogeneic hematopoietic stem cell transplantation, Auto-HSCT: Autologous hematopoietic stem cell transplantation, chrt: chemotherapy, MDS: Myelodysplastic syndrom, FAB: French-American-British, CBF: Core binding factor leukemia, ASXL1: additional sex combs like 1, CEBPA: CCAAT/enhancer binding protein alpha, DNMT3A: DNA (cytosine-5-)-methyltransferase 3 alpha, FLT3-TKD: fms related tyrosine kinase 3-tyrosine kinase domain mutation, IDH1: isocitrate dehydrogenase (NADP(+)) 1, IDH2: isocitrate dehydrogenase (NADP(+)) 2, KRAS: Kirsten Rat Sarcoma Viral Proto-Oncogene, NPM1: nucleophosmin 1, NRAS: Neuroblastoma RAS Viral Oncogene Homolog. * indicates groups of p-values of which the false discovery rate (fdr) is calulated. | | | | | | |  |

| **Cohort 2 (FLT3-ITD t-VAF </≥ 0.7)** | | | | | | |  |
| --- | --- | --- | --- | --- | --- | --- | --- |
| **ST5-C2** | **<0.7** | | **≥0.7** | | **P-Value** | **FDR adj.** |  |
| Clinical parameters | |  |  |  |  |  |  |
| Age | 50 | (103/103) | 53.5 | (20/20) | 0.626 | 0.626 | * |
| PLT | 64 | (103/103) | 61.5 | (20/20) | 0.513 | 0.626 |  |
| WBC | 36 | (103/103) | 62.9 | (20/20) | 0.030 | 0.090 |  |
| Female | 54/103 | (52.4%) | 12/20 | (60%) | 0.628 |  |  |
| Transplantation |  |  |  |  |  |  |  |
| Allo-HSCT | 73/103 | (70.9%) | 9/20 | (45%) | **0.037** |  |  |
| Auto-HSCT | 10/103 | (9.7%) | 3/20 | (15%) | 0.442 |  |  |
| Predisposition |  |  |  |  |  |  |  |
| Prior chrt | 5/99 | (5.1%) | 0/20 | (0%) | 0.588 |  |  |
| Prior disease | 7/103 | (6.8%) | 0/20 | (0%) | 0.597 |  |  |
| Prior ins | 0/68 | (0%) | 0/16 | (0%) | 1.000 |  |  |
| Prior MDS | 5/102 | (4.9%) | 1/20 | (5%) | 1.000 |  |  |
| FAB classification |  |  |  |  |  |  |  |
| M0 | 6/100 | (6%) | 0/19 | (0%) | 0.588 | 1.000 | * |
| M1 | 32/100 | (32%) | 7/19 | (36.8%) | 0.791 | 1.000 |  |
| M2 | 30/100 | (30%) | 6/19 | (31.6%) | 1.000 | 1.000 |  |
| M4 | 8/100 | (8%) | 0/19 | (0%) | 0.352 | 1.000 |  |
| M5 | 21/100 | (21%) | 5/19 | (26.3%) | 0.561 | 1.000 |  |
| M6 | 3/100 | (3%) | 0/19 | (0%) | 1.000 | 1.000 |  |
| M7 | 0/100 | (0%) | 1/19 | (5.3%) | 0.160 | 1.000 |  |
| Karyotype |  |  |  |  |  |  |  |
| CBF | 4/103 | (3.9%) | 0/20 | (0%) | 1.000 | 1.000 | * |
| Complex | 1/103 | (1%) | 0/20 | (0%) | 1.000 | 1.000 |  |
| inv(16) | 1/103 | (1%) | 0/20 | (0%) | 1.000 | 1.000 |  |
| Normal | 75/103 | (72.8%) | 16/20 | (80%) | 0.589 | 1.000 |  |
| + 8 | 0/75 | (0%) | 0/16 | (0%) | 1.000 | 1.000 |  |
| t(8:21) | 3/103 | (2.9%) | 0/20 | (0%) | 1.000 | 1.000 |  |
| Mutation status |  |  |  |  |  |  |  |
| ASXL1 | 3/98 | (3.1%) | 0/19 | (0%) | 1.000 | 1.000 | * |
| CEBPA double | 4/85 | (4.7%) | 0/16 | (0%) | 1.000 | 1.000 |  |
| DNMT3A | 38/98 | (38.8%) | 16/19 | (84.2%) | 0.000 | **0.005** |  |
| FLT3-TKD | 10/99 | (10.1%) | 1/19 | (5.3%) | 1.000 | 1.000 |  |
| IDH1 | 9/98 | (9.2%) | 2/19 | (10.5%) | 1.000 | 1.000 |  |
| IDH2 | 19/98 | (19.4%) | 2/19 | (10.5%) | 0.519 | 1.000 |  |
| JAK2 | 0/98 | (0%) | 0/19 | (0%) | 1.000 | 1.000 |  |
| NPM1 | 51/103 | (49.5%) | 16/20 | (80%) | 0.014 | 0.107 |  |
| PTPN11 | 8/98 | (8.2%) | 0/19 | (0%) | 0.351 | 1.000 |  |
| RUNX1 | 13/98 | (13.3%) | 0/19 | (0%) | 0.123 | 0.617 |  |
| SF3B1 | 3/98 | (3.1%) | 0/19 | (0%) | 1.000 | 1.000 |  |
| SRSF2 | 4/98 | (4.1%) | 0/19 | (0%) | 1.000 | 1.000 |  |
| TET2 | 12/98 | (12.2%) | 0/19 | (0%) | 0.211 | 0.790 |  |
| TP53 | 1/98 | (1%) | 0/19 | (0%) | 1.000 | 1.000 |  |
| WT1 | 15/98 | (15.3%) | 4/19 | (21.1%) | 0.509 | 1.000 |  |
|  |  |  |  |  |  |  |  |
| PLT: Platelet count (10^9^/L), WBC: White blood cell count (10^9^/L), Allo-HSCT: Allogeneic hematopoietic stem cell transplantation, Auto-HSCT: Autologous hematopoietic stem cell transplantation, chrt: chemotherapy, ins: insecticide, MDS: Myelodysplastic syndrom, FAB: French-American-British, CBF: Core binding factor leukemia, ASXL1: additional sex combs like 1, CEBPA: CCAAT/enhancer binding protein alpha, DNMT3A: DNA (cytosine-5-)-methyltransferase 3 alpha, FLT3-TKD: fms related tyrosine kinase 3-tyrosine kinase domain mutation, IDH1: isocitrate dehydrogenase (NADP(+)) 1, IDH2: isocitrate dehydrogenase (NADP(+)) 2, KRAS: Kirsten Rat Sarcoma Viral Proto-Oncogene, NPM1: nucleophosmin 1, NRAS: Neuroblastoma RAS Viral Oncogene Homolog, JAK2: Janus kinase 2, PTPN11: Tyrosine-protein phosphatase non-receptor type 11, RUNX1: Runt-related transcription factor 1, SF3B1: Splicing factor 3B subunit 1, SRSF2: Serine And Arginine Rich Splicing Factor 2, TET2: Tet methylcytosine dioxygenase 2, TP53: Tumor protein p53, WT1: Wilms tumor protein. * indicates groups of p-values of which the false discovery rate (fdr) is calulated. | | | | | | |  |

| **Cohort 1 (Short versus long FLT3-ITD major)** | | | | | | |  |
| --- | --- | --- | --- | --- | --- | --- | --- |
| **ST6-C1** | **Short ITD (<50 bp)** | | **Long ITD (≥50 bp)** | | **P-Value** | **FDR adj.** |  |
| Clinical parameters | |  |  |  |  |  |  |
| Age | 45 | (55/55) | 47.5 | (56/56) | 0.382 | 0.491 | * |
| BM blast % | 78.5 | (54/55) | 82.5 | (54/56) | 0.491 | 0.491 |  |
| PLT | 57 | (55/55) | 67.5 | (56/56) | 0.348 | 0.491 |  |
| WBC | 57.8 | (55/55) | 68.8 | (56/56) | 0.381 | 0.491 |  |
| Female | 34/55 | (61.8%) | 31/56 | (55.4%) | 0.565 |  |  |
| Transplantation |  |  |  |  |  |  |  |
| Allo-HSCT | 15/55 | (27.3%) | 12/56 | (21.4%) | 0.513 |  |  |
| Auto-HSCT | 8/55 | (14.5%) | 7/56 | (12.5%) | 0.788 |  |  |
| Predisposition |  |  |  |  |  |  |  |
| Prior chrt | 0/55 | (0%) | 0/56 | (0%) | 1.000 |  |  |
| Prior MDS | 1/55 | (1.8%) | 1/56 | (0%) | 1.000 |  |  |
| FAB classification |  |  |  |  |  |  |  |
| M0 | 0/55 | (0%) | 1/56 | (1.8%) | 1.000 | 1.000 | * |
| M1 | 16/55 | (29.1%) | 16/56 | (28.6%) | 1.000 | 1.000 |  |
| M2 | 14/55 | (25.5%) | 12/56 | (21.4%) | 0.659 | 1.000 |  |
| M4 | 4/55 | (7.3%) | 11/56 | (19.6%) | 0.094 | 0.564 |  |
| M5 | 21/55 | (38.2%) | 16/56 | (28.6%) | 0.318 | 0.954 |  |
| M6 | 0/55 | (0%) | 0/56 | (0%) | 1.000 | 1.000 |  |
| Karyotype |  |  |  |  |  |  |  |
| CBF | 2/55 | (3.6%) | 1/56 | (1.8%) | 0.618 | 1.000 | * |
| Complex | 0/55 | (0%) | 1/56 | (1.8%) | 1.000 | 1.000 |  |
| inv(16) | 0/55 | (0%) | 0/55 | (0%) | 1.000 | 1.000 |  |
| Normal | 42/55 | (76.4%) | 39/56 | (69.6%) | 0.666 | 1.000 |  |
| + 8 | 3/55 | (5.5%) | 4/55 | (7.3%) | 1.000 | 1.000 |  |
| t(6:9) | 1/55 | (1.8%) | 2/55 | (3.6%) | 1.000 | 1.000 |  |
| t(8:21) | 2/55 | (3.6%) | 1/55 | (1.8%) | 0.618 | 1.000 |  |
| t(9:22) | 0/55 | (0%) | 0/56 | (0%) | 1.000 | 1.000 |  |
| Mutation status |  |  |  |  |  |  |  |
| ASXL1 | 1/55 | (1.8%) | 0/55 | (0%) | 0.495 | 1.000 | * |
| CEBPA | 3/55 | (5.5%) | 4/56 | (7.1%) | 1.000 | 1.000 |  |
| CEBPA-double | 2/55 | (3.6%) | 1/56 | (1.8%) | 0.618 | 1.000 |  |
| DNMT3A | 25/55 | (45.5%) | 10/56 | (17.9%) | 0.002 | **0.022** |  |
| FLT3-TKD | 3/55 | (5.5%) | 3/56 | (5.4%) | 1.000 | 1.000 |  |
| IDH1 | 7/55 | (12.7%) | 2/56 | (3.6%) | 0.094 | 0.312 |  |
| IDH2 | 2/55 | (3.6%) | 2/56 | (3.6%) | 1.000 | 1.000 |  |
| KRAS | 0/55 | (0%) | 0/56 | (0%) | 1.000 | 1.000 |  |
| NPM1 | 40/55 | (72.7%) | 29/56 | (51.8%) | 0.031 | 0.156 |  |
| NRAS | 0/55 | (0%) | 0/56 | (0%) | 1.000 | 1.000 |  |
|  |  |  |  |  |  |  |  |
| BM blast %: Bone marrow blast percentage, PLT: Platelet count (10^9^/L), WBC: White blood cell count (10^9^/L), Allo-HSCT: Allogeneic hematopoietic stem cell transplantation, Auto-HSCT: Autologous hematopoietic stem cell transplantation, chrt: chemotherapy, MDS: Myelodysplastic syndrom, FAB: French-American-British, CBF: Core binding factor leukemia, ASXL1: additional sex combs like 1, CEBPA: CCAAT/enhancer binding protein alpha, DNMT3A: DNA (cytosine-5-)-methyltransferase 3 alpha, FLT3-TKD: fms related tyrosine kinase 3-tyrosine kinase domain mutation, IDH1: isocitrate dehydrogenase (NADP(+)) 1, IDH2: isocitrate dehydrogenase (NADP(+)) 2, KRAS: Kirsten Rat Sarcoma Viral Proto-Oncogene, NPM1: nucleophosmin 1, NRAS: Neuroblastoma RAS Viral Oncogene Homolog. * indicates groups of p-values of which the false discovery rate (fdr) is calulated. | | | | | | |  |

| **Cohort 2 (Short versus long FLT3-ITD major)** | | | | | | |  |
| --- | --- | --- | --- | --- | --- | --- | --- |
| **ST6-C2** | **Short ITD (<50 bp)** | | **Long ITD (≥50 bp)** | | **P-Value** | **FDR adj.** |  |
| **Clinical parameters** | |  |  |  |  |  |  |
| Age | 51 | (66/66) | 49 | (57/57) | 0.788 | 0.788 | * |
| PLT | 59 | (66/66) | 68 | (57/57) | 0.105 | 0.315 |  |
| WBC | 38.9 | (66/66) | 48.3 | (57/57) | 0.261 | 0.392 |  |
| Female | 36/66 | (54.5%) | 30/57 | (52.6%) | 0.858 |  |  |
| Transplantation |  |  |  |  |  |  |  |
| Allo-HSCT | 46/66 | (69.7%) | 36/57 | (63.2%) | 0.451 |  |  |
| Auto-HSCT | 8/66 | (12.1%) | 5/57 | (8.8%) | 0.770 |  |  |
| Predisposition |  |  |  |  |  |  |  |
| Prior chrt | 3/63 | (4.8%) | 2/56 | (3.6%) | 1.000 |  |  |
| Prior disease | 1/66 | (1.5%) | 6/57 | (10.5%) | **0.048** |  |  |
| Prior ins | 0/47 | (0%) | 0/37 | (0%) | 1.000 |  |  |
| Prior MDS | 2/66 | (3%) | 4/56 | (7.1%) | 0.412 |  |  |
| FAB classification |  |  |  |  |  |  |  |
| M0 | 1/65 | (1.5%) | 5/54 | (9.3%) | 0.090 | 0.633 | * |
| M1 | 23/65 | (35.4%) | 16/54 | (29.6%) | 0.560 | 1.000 |  |
| M2 | 20/65 | (30.8%) | 16/54 | (29.6%) | 1.000 | 1.000 |  |
| M4 | 5/65 | (7.7%) | 3/54 | (5.6%) | 0.727 | 1.000 |  |
| M5 | 14/65 | (21.5%) | 12/54 | (22.2%) | 1.000 | 1.000 |  |
| M6 | 2/65 | (3.1%) | 1/54 | (1.9%) | 1.000 | 1.000 |  |
| M7 | 0/65 | (0%) | 1/54 | (1.9%) | 0.454 | 1.000 |  |
| Karyotype |  |  |  |  |  |  |  |
| CBF | 4/66 | (6.1%) | 0/57 | (0%) | 0.123 | 0.615 | * |
| Complex | 0/66 | (0%) | 1/57 | (1.8%) | 0.463 | 0.772 |  |
| inv(16) | 1/66 | (1.5%) | 0/57 | (0%) | 1.000 | 1.000 |  |
| Normal | 50/66 | (75.8%) | 41/57 | (71.9%) | 0.683 | 0.854 |  |
| t(8:21) | 3/66 | (4.5%) | 0/57 | (0%) | 0.248 | 0.620 |  |
| Mutation status |  |  |  |  |  |  |  |
| ASXL1 | 1/64 | (1.6%) | 2/53 | (3.8%) | 0.589 | 0.853 | * |
| CEBPA double | 1/57 | (1.8%) | 3/44 | (6.8%) | 0.315 | 0.640 |  |
| DNMT3A | 29/64 | (45.3%) | 25/53 | (47.2%) | 0.854 | 0.986 |  |
| FLT3-TKD | 4/64 | (6.3%) | 7/54 | (13%) | 0.341 | 0.640 |  |
| IDH1 | 8/64 | (12.5%) | 3/53 | (5.7%) | 0.341 | 0.640 |  |
| IDH2 | 15/64 | (23.4%) | 6/53 | (11.3%) | 0.098 | 0.640 |  |
| JAK2 | 0/64 | (0%) | 0/53 | (0%) | 1.000 | 1.000 |  |
| NPM1 | 39/66 | (59.1%) | 28/57 | (49.1%) | 0.282 | 0.640 |  |
| PTPN11 | 5/64 | (7.8%) | 3/53 | (5.7%) | 0.727 | 0.909 |  |
| RUNX1 | 5/64 | (7.8%) | 8/53 | (15.1%) | 0.247 | 0.640 |  |
| SF3B1 | 0/64 | (0%) | 3/53 | (5.7%) | 0.090 | 0.640 |  |
| SRSF2 | 3/64 | (4.7%) | 1/53 | (1.9%) | 0.625 | 0.853 |  |
| TET2 | 8/64 | (12.5%) | 4/53 | (7.5%) | 0.543 | 0.853 |  |
| TP53 | 1/64 | (1.6%) | 0/53 | (0%) | 1.000 | 1.000 |  |
| WT1 | 7/64 | (10.9%) | 12/53 | (22.6%) | 0.130 | 0.640 |  |
|  |  |  |  |  |  |  |  |
| PLT: Platelet count (10^9^/L), WBC: White blood cell count (10^9^/L), Allo-HSCT: Allogeneic hematopoietic stem cell transplantation, Auto-HSCT: Autologous hematopoietic stem cell transplantation, chrt: chemotherapy, ins: insecticide, MDS: Myelodysplastic syndrom, FAB: French-American-British, CBF: Core binding factor leukemia, ASXL1: additional sex combs like 1, CEBPA: CCAAT/enhancer binding protein alpha, DNMT3A: DNA (cytosine-5-)-methyltransferase 3 alpha, FLT3-TKD: fms related tyrosine kinase 3-tyrosine kinase domain mutation, IDH1: isocitrate dehydrogenase (NADP(+)) 1, IDH2: isocitrate dehydrogenase (NADP(+)) 2, KRAS: Kirsten Rat Sarcoma Viral Proto-Oncogene, NPM1: nucleophosmin 1, NRAS: Neuroblastoma RAS Viral Oncogene Homolog, JAK2: Janus kinase 2, PTPN11: Tyrosine-protein phosphatase non-receptor type 11, RUNX1: Runt-related transcription factor 1, SF3B1: Splicing factor 3B subunit 1, SRSF2: Serine And Arginine Rich Splicing Factor 2, TET2: Tet methylcytosine dioxygenase 2, TP53: Tumor protein p53, WT1: Wilms tumor protein. * indicates groups of p-values of which the false discovery rate (fdr) is calulated. | | | | | | |  |

| **ST7-C1**  **Survival Analysis Cohort 1 (n = 111)** | | | | | |
| --- | --- | --- | --- | --- | --- |
| **Time (Months)** | **Number at risk** | **Number of events** | **Survival** | **standard error** | **95% CI** |
| LM1 <50 | | | | | |
| Median | 55 | 35 | 15.21 months |  | (9.20 - NA) |
| 12 | 31 | 24 | 0.564 | 0.0669 | (0.447 - 0.711) |
| 24 | 23 | 8 | 0.418 | 0.0665 | (0.306 - 0.571) |
| 36 | 20 | 2 | 0.381 | 0.0656 | (0.272 - 0.534) |
| 48 | 19 | 1 | 0.362 | 0.065 | (0.254 - 0.515) |
| 60 | 17 | 0 | 0.362 | 0.065 | (0.254 - 0.515) |
|  |  |  |  |  |  |
| LM1 ≥50 | | | | | |
| Median | 56 | 48 | 7.66 months |  | (6.93 - 14.2) |
| 12 | 20 | 36 | 0.357 | 0.064 | (0.2513 - 0.508) |
| 24 | 13 | 7 | 0.232 | 0.0564 | (0.1442 - 0.374) |
| 36 | 12 | 1 | 0.214 | 0.0548 | (0.1298 - 0.354) |
| 48 | 10 | 1 | 0.196 | 0.0531 | (0.1156 - 0.334) |
| 60 | 4 | 2 | 0.135 | 0.0516 | (0.0636 - 0.285) |
|  |  |  |  |  |  |
| t-VAF <0.7 | | | | | |
| Median | 84 | 60 | 13.5 months |  | (8.25-24.1) |
| 12 | 44 | 40 | 0.524 | 0.0545 | (0.427 - 0.642) |
| 24 | 32 | 12 | 0.381 | 0.053 | (0.29 - 0.5) |
| 36 | 28 | 3 | 0.345 | 0.0519 | (0.257 - 0.463) |
| 48 | 26 | 2 | 0.32 | 0.051 | (0.234 - 0.438) |
| 60 | 18 | 2 | 0.29 | 0.0505 | (0.207 - 0.408) |
|  |  |  |  |  |  |
| t-VAF ≥0.7 | | | | | |
| Median | 27 | 23 | 8.11 months |  | (6.51 - 17.0) |
| 12 | 7 | 20 | 0.259 | 0.0843 | (0.137 - 0.49) |
| 24 | 4 | 3 | 0.148 | 0.0684 | (0.06 - 0.366) |
| 36 | 4 | 0 | 0.148 | 0.0684 | (0.06 - 0.366) |
| 48 | 3 | 0 | 0.148 | 0.0684 | (0.06 - 0.366) |
| 60 | 3 | 0 | 0.148 | 0.0684 | (0.06 - 0.366) |
|  |  |  |  |  |  |
| Plural FLT3-ITDs | | | | | |
| Median | 24 | 15 | 14.6 months |  | (7.23 - NA) |
| 12 | 13 | 11 | 0.542 | 0.102 | (0.375 - 0.783) |
| 24 | 11 | 2 | 0.458 | 0.102 | (0.297 - 0.708) |
| 36 | 11 | 0 | 0.458 | 0.102 | (0.297 - 0.708) |
| 48 | 10 | 1 | 0.417 | 0.101 | (0.26 - 0.669) |
| 60 | 5 | 1 | 0.357 | 0.102 | (0.204 - 0.626) |
|  |  |  |  |  |  |
| Single FLT3-ITDs | | | | | |
| Median | 87 | 68 | 8.9 months |  | (7.98 - 15.2) |
| 12 | 38 | 49 | 0.437 | 0.0532 | (0.344 - 0.554) |
| 24 | 25 | 13 | 0.287 | 0.0485 | (0.206 - 0.4) |
| 36 | 21 | 3 | 0.252 | 0.0466 | (0.176 - 0.362) |
| 48 | 19 | 1 | 0.24 | 0.0459 | (0.165 - 0.35) |
| 60 | 16 | 1 | 0.227 | 0.0453 | (0.154 - 0.336) |
|  |  |  |  |  |  |

| **ST7-C2**  **Survival analysis Cohort 2 (n = 123)** | | | | | |
| --- | --- | --- | --- | --- | --- |
| **Time (Months)** | **Number at risk** | **Number of events** | **Survival** | **standard error** | **95% CI** |
| LM1 <50 | | | | | |
| Median | 66 | 35 | 26.6 months |  | (13.7 - NA) |
| 12 | 39 | 25 | 0.618 | 0.0601 | (0.511 - 0.748) |
| 24 | 31 | 7 | 0.506 | 0.0624 | (0.397 - 0.644) |
| 36 | 19 | 2 | 0.471 | 0.0629 | (0.363 - 0.612) |
| 48 | 11 | 1 | 0.44 | 0.066 | (0.328 - 0.59) |
| 60 | 8 | 0 | 0.44 | 0.066 | (0.328 - 0.59) |
|  |  |  |  |  |  |
| LM1 ≥50 | | | | | |
| Median | 57 | 38 | 14.6 months |  | (10.30-30.0) |
| 12 | 32 | 25 | 0.561 | 0.0657 | (0.446 - 0.706) |
| 24 | 22 | 9 | 0.403 | 0.0651 | (0.293 - 0.553) |
| 36 | 15 | 3 | 0.347 | 0.0636 | (0.242 - 0.497) |
| 48 | 5 | 1 | 0.297 | 0.0712 | (0.186 - 0.475) |
| 60 | 1 | 0 | 0.297 | 0.0712 | (0.186 - 0.475) |
|  |  |  |  |  |  |
| t-VAF <0.7 | | | | | |
| Median | 103 | 56 | 26.6 months |  | (14.6 - NA) |
| 12 | 66 | 35 | 0.658 | 0.0469 | (0.572 - 0.757) |
| 24 | 49 | 15 | 0.507 | 0.0498 | (0.418 - 0.615) |
| 36 | 32 | 4 | 0.463 | 0.0501 | (0.375 - 0.573) |
| 48 | 14 | 2 | 0.418 | 0.0548 | (0.323 - 0.54) |
| 60 | 7 | 0 | 0.418 | 0.0548 | (0.323 - 0.54) |
|  |  |  |  |  |  |
| t-VAF ≥0.7 | | | | | |
| Median | 20 | 17 | 7.8 months |  | (7 - 17.0) |
| 12 | 5 | 15 | 0.25 | 0.0968 | (0.117 - 0.534) |
| 24 | 4 | 1 | 0.2 | 0.0894 | (0.0832 - 0.481) |
| 36 | 2 | 1 | 0.15 | 0.0798 | (0.0528 - 0.426) |
| 48 | 2 | 0 | 0.15 | 0.0798 | (0.0528 - 0.426) |
| 60 | 2 | 0 | 0.15 | 0.0798 | (0.0528 - 0.426) |
|  |  |  |  |  |  |
| Plural FLT3-ITDs | | | | | |
| Median | 35 | 20 | 14.6 months |  | (8.6 - NA) |
| 12 | 17 | 16 | 0.533 | 0.0858 | (0.388 - 0.73) |
| 24 | 12 | 4 | 0.407 | 0.0855 | (0.27 - 0.615) |
| 36 | 7 | 0 | 0.407 | 0.0855 | (0.27 - 0.615) |
| 48 | 5 | 0 | 0.407 | 0.0855 | (0.27 - 0.615) |
| 60 | 3 | 0 | 0.407 | 0.0855 | (0.27 - 0.615) |
|  |  |  |  |  |  |
| Single FLT3-ITDs | | | | | |
| Median | 88 | 53 | 20.8 months |  | (13.9-45.5) |
| 12 | 54 | 34 | 0.614 | 0.0519 | (0.52 - 0.724) |
| 24 | 41 | 12 | 0.476 | 0.0534 | (0.382 - 0.593) |
| 36 | 27 | 5 | 0.415 | 0.053 | (0.323 - 0.533) |
| 48 | 11 | 2 | 0.367 | 0.0571 | (0.271 - 0.498) |
| 60 | 6 | 0 | 0.367 | 0.0571 | (0.271 - 0.498) |
|  |  |  |  |  |  |

| **ST8-C1**  **Cox Regression Analysis, Cohort 1 (n= 111, number of events= 83)** | | | | | | | | | |
| --- | --- | --- | --- | --- | --- | --- | --- | --- | --- |
|  | **Univariate analysis** | |  | | | | **Multivariate analysis** | | |
|  | HR (95% CI for HR) | p-value |  | | | | HR (95% CI for HR) | | p-value |
| Age | 1 (1-1.03) | 0.1596 |  | | | | 1.01 (0.99 - 1.03) | | 0.1932 |
| WBC | 1 (1-1) | **0.02526** |  | | | | 1.00 (1 - 1) | | 0.5893 |
| Female | 1.4 (0.92-2.25) | 0.1128 |  | | | | 1.63 (1.02 - 2.61) | | **0.0413** |
| Plural FLT3-ITDs | 0.72 (0.41-1.25) | 0.2418 |  | | | | 1.21 (0.68 - 2.14) | | 0.5165 |
| LM1 length | 1 (1-1.02) | **0.02788** |  | | | | 1.01 (1 - 1.02) | | 0.0614 |
| t-VAF | 1.9 (0.83-4.46) | 0.1277 |  | | | | 1.81 (0.69 - 4.71) | | 0.2245 |
|  |  | | |  |  |  |  |  |  |
|  |  | | |  |  |  |  |  |  |
|  |  | | |  |  |  |  |  |  |
| **ST8-C2**  **Cox Regression Analysis, Cohort 2 ( n= 123, number of events= 73 )** | | | | | | | | | |
|  | **Univariate analysis** | |  | | | | **Multivariate analysis** | | |
|  | HR (95% CI for HR) | p-value |  | | | | HR (95% CI for HR) | | p-value |
| Age | 1 (0.99-1) | 0.28 |  | | | | 1.01 (0.99 - 1.04) | | 0.2142 |
| WBC | 1 (1-1) | **0.028** |  | | | | 1.00 (1 - 1.01) | | 0.0674 |
| Female | 1.1 (0.72-1.8) | 0.59 |  | | | | 0.77 (0.48 - 1.23) | | 0.271 |
| Plural FLT3-ITDs | 1.1 (0.64-1.8) | 0.79 |  | | | | 1.01 (0.6 - 1.72) | | 0.9683 |
| LM1 length | 1 (0.99-1) | 0.88 |  | | | | 1 (1 - 1.01) | | 0.9329 |
| t-VAF | 3.9 (1.6-9.5) | **0.0025** |  | | | | 3.59 (1.45 - 8.89) | | **0.0057** |
|  |  | | |  |  |  |  |  |  |
